# Electrotherapies for Glioblastoma

**DOI:** 10.1002/advs.202100978

**Published:** 2021-07-22

**Authors:** Elise P. W. Jenkins, Alina Finch, Magda Gerigk, Iasonas F. Triantis, Colin Watts, George G. Malliaras

**Affiliations:** ^1^ Division of Electrical Engineering Department of Engineering University of Cambridge Cambridge CB3 0FA UK; ^2^ Institute of Cancer and Genomic Science University of Birmingham Birmingham B15 2TT UK; ^3^ Department of Electrical and Electronic Engineering City, University of London London EC1V 0HB UK

**Keywords:** bioelectronics, electroporation, electrotherapy, glioblastoma, tumour‐treating fields

## Abstract

Non‐thermal, intermediate frequency (100–500 kHz) electrotherapies present a unique therapeutic strategy to treat malignant neoplasms. Here, pulsed electric fields (PEFs) which induce reversible or irreversible electroporation (IRE) and tumour‐treating fields (TTFs) are reviewed highlighting the foundations, advances, and considerations of each method when applied to glioblastoma (GBM). Several biological aspects of GBM that contribute to treatment complexity (heterogeneity, recurrence, resistance, and blood‐brain barrier(BBB)) and electrophysiological traits which are suggested to promote glioma progression are described. Particularly, the biological responses at the cellular and molecular level to specific parameters of the electrical stimuli are discussed offering ways to compare these parameters despite the lack of a universally adopted physical description. Reviewing the literature, a disconnect is found between electrotherapy techniques and how they target the biological complexities of GBM that make treatment difficult in the first place. An attempt is made to bridge the interdisciplinary gap by mapping biological characteristics to different methods of electrotherapy, suggesting important future research topics and directions in both understanding and treating GBM. To the authors' knowledge, this is the first paper that attempts an in‐tandem assessment of the biological effects of different aspects of intermediate frequency electrotherapy methods, thus offering possible strategies toward GBM treatment.

## Graphical abstract

Electrotherapy uses electric fields to target various biological mechanisms for therapeutic outcomes. The current state of intermediate frequency electric fields including tumour‐treating fields, and reversible/irreversible electroporation are discussed in both pre‐clinical and clinical models of glioblastoma. Where possible, these techniques are mapped to the complexities of the disease and opportunities and directions for future research are highlighted.

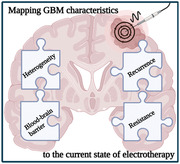

## 1 Introduction

Glioblastoma (GBM), a type of brain tumour, is an aggressive, debilitating disease with poor prognosis and limited changes to the standard of care since 2005 despite substantial research efforts.^[^ ^1^ ^]^ Currently, surgical resection where possible followed by radiotherapy and adjuvant chemotherapy,Temozolomide (TMZ)is often considered as the optimal treatment strategy. Radiotherapy (RT), also known as radiation therapy, uses high energy electromagnetic fields or radioactive substances to directly or indirectly damage DNA of tumour cells to halt or slow tumour growth.^[^ ^2^ ^]^ Temozolomide (TMZ) is an alkylating agent approved for anti‐cancer treatment of GBM that is capable of crossing the blood‐brain barrier (BBB). Simply, it aims to damage DNA and trigger cell suicide.^[^ ^3^ ^]^ However, depending on the expression of the MGMT (O‐6‐methylguanine‐DNA methyltransferase) gene, GBM cells can repair the DNA damage and become resistant to treatment resulting in poor therapeutic efficacy and inevitable recurrence.^[^ ^3^ ^]^ Recent efforts that focus on cancer drug delivery techniques to overcome the BBB include encapsulated nanoparticles, drug delivery vehicles, convection enhanced delivery,^[^ ^4^ ^]^ and immunotherapy strategies to enhance T‐cell infiltration.^[^ ^5^ ^]^ Despite these efforts, the complexity of the disease results in treatment failure, disease progression and fatality.

While pharmaceutical interventions, as well as, the study of chemical and mechanical aspects of biology and medicine have been the focus of therapeutic and diagnostic research and practice since antiquity, only glimpses of the therapeutic effects of electricity were documented early on, notably through the use of electric fish for pain relief by Scribonius Largus circa 47CE.^[^ ^6^ ^]^ It is mainly in the last few centuries that bioelectric aspects of diagnosis and therapy have been considered, the first through biology's electrical properties and signals and the latter through electrical and electromagnetic interventions.^[^ ^7^ ^]^ Electrical stimulation via bespoke apparatus was demonstrated by Galvani over 200 years ago, causing frog legs to twitch. The electrophysiology era continued well into the 1800s to date where scientific breakthroughs led to auditory, visual, olfactory stimulation, cochlear implants, cardiac pacemakers, functional electrical stimulation and deep brain stimulation.^[^ ^8^ ^]^ Akin to pharmaceutical interventions, being subject to dosage quantity, timing and frequency as well as application route and location, the efficacy of electrical interventions depends on the waveform, amplitude and frequency of the applied signal as the topological aspects of the active electrodes. Alternating current (AC) stimuli in the low frequency spectrum <10 kHz when applied to living tissue can excite cells by depolarizing the cell membrane. Increasing the applied frequency beyond 10 kHz toward a few MHz (herein referred to as intermediate frequencies) were long believed to have insignificant cellular effects. At higher frequencies, tissue heating phenomena are observed, and this is exploited in many tissue ablation techniques, most notably, radio‐frequency tumour ablation.

**Figure 1:**
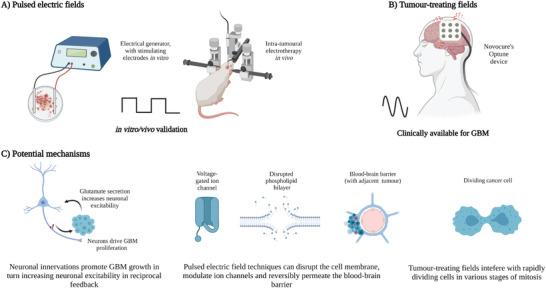
Stages and potential biological modulation of electrotherapies. A) Pulsed electric fields (including nanosecond‐pulsed electric fields (nsPEFs), reversible electroporation and IRE techniques) targeting GBM are in pre‐clinical validation with most studies in vitro and others in canine and rat models. These techniques typically involve mono‐ or bi‐phasic waveforms. B) TTFs is FDA approved to treat new and recurrent GBM in combination with current standard of care. It typically involves sinusoidal AC fields at 200 kHz. C) Potential mechanisms (from left to right): Neuron signaling contributes to GBM growth which feeds back to increase neuron excitation. PEF techniques can target ion channels, lipid bilayers, blood‐brain barrier opening, and other intracellular mechanisms (not shown). TTFs target mitotic cells through dipoles and DEP forces. Created with BioRender.com

For the purpose of establishing nomenclature, applied voltages are herein denoted by *V* (in Volts) and electric fields by ***E*** (Volts cm^−1^). In electrotherapy (see **Figure** **1**), non‐thermal intermediate frequencies treatments include pulsed‐electric fields (PEFs) which range from nanosecond to millisecond pulsing durations and tumour‐treating fields (TTFs) ranging from 100 to 500 kHz. PEFs can be used to induce electroporation,^[^ ^9^ ^]^ a technique whereby electrodes are placed within target tissue to induce nano‐sized pores in cell membranes, increasing their permeability and thus susceptibility of drug substances (e.g., for electrochemotherapy (ECT)^[^ ^10^ ^]^) or delivery of DNA (electrogenetherapy^[^ ^11^ ^]^) allowing for targeted pharmaceutical intervention. When the applied field ***E*** is substantial (i.e., 1 kV cm^−1^), irreversible breakdown of the cell membrane occurs leading to cell death (IRE).^[^ ^12^ ^]^ Alternatively, TTFs are non‐invasive and use fields of ***E*** ≈ 1–3 V cm^−1^ which are claimed to interfere with mitosis (although this is disputed as indicated later) where dipole molecules like tubulin dimers of microtubules align with the applied field leading to improper polymerization and early metaphase exit. In addition, cells in the final stage of mitosis (telophase) are subject to non‐uniform ***E*** that create dielectrophoretic (DEP) forces moving particles toward the furrow prompting DNA damage and cellular suicide.^[^ ^13^, ^14^, ^15^ ^]^ In the treatment of GBM, electroporation techniques are still in pre‐clinical stages, while TTFs are Food and Drug Administration (FDA) approved for newly diagnosed and recurrent disease. These methods have shown very promising results, with electroporation mostly demonstrated to affect cancerous cells pre‐clinically,^[^ ^16^ ^]^ while TTFs has been approved for clinical use to treat GBM (NCT00916409). Still, the first is not yet fully proven for GBM whilst the latter is not fully accepted by the medical community, mainly due to the lack of understanding of its underlying mechanisms and controversial clinical trial data.^[^ ^17^ ^]^ Both techniques target specific aspects of the tumour and thus they are challenged by the multi‐faceted nature of GBM much like the challenges faced in bespoke pharmaceutical remedies.

Therefore, there is a need for better understanding of the effect of each of the aforementioned methods to biological mechanisms that have a direct remedial impact on GBM. To be more precise, there is a need for “mapping” the effects of each of the reported stimulus parameters of these techniques to corresponding biological aspects of GBM. This is particularly challenging due to the increased biological complexity related to the poorly understood “multiforme” or heterogeneous nature of GBM tumors,^[^ ^18^, ^19^ ^]^ as described later. As the authors have noticed, this is coupled with an additional difficulty from the lack of consistency in literature when it comes to describing the physical characteristics of the applied electrotherapeutic signal. For example, some papers mentioning field strength of extracranial fields while others report intracranial fields (i.e., volume conductors,^[^ ^7^ ^]^ or applied voltage or current amplitudes) without a clear “translation” from one to the other, rendering certain studies incomparable. Additional inconsistencies come from differences in electrode topologies, as well as their material or their proximity to the targeted tumour.

Finally, given the biophysical understanding of the cell membrane potential and electrophysiology, it seems intuitive that electrotherapy techniques could exploit the electrical characteristics of glioma. While this may be the case, it appears there is an interdisciplinary disconnect at the interface of neuroscience, biology, and applied physics/engineering that is limiting the pace of progression in this field, thus, we propose research questions that require urgent consideration.

## 2 Addressing the Biological Challenge

### 2.1 Glioblastoma Multiforme

The central nervous system (CNS) is built up of neurons and glial cells, with the latter primarily serving the needs of the first by providing scaffolds during the development of the nervous system.^[^ ^20^, ^21^ ^]^ Glia can also produce brief electric currents by opening calcium (Ca^2+^) channels influencing many neurons almost simultaneously and affecting neurotransmitter release, hence contribute to the coordination of synaptic activity.^[^ ^20^, ^21^, ^22^, ^23^ ^]^ Glial cells are usually divided into three categories: astrocytes, oligodendrocytes and microglial cells. Astrocytes exhibit a variety of shapes and appear to serve a homeostatic function. They also surround blood capillaries and form very extensive tight junctions between endothelial cells, thus decreasing the permeability of brain capillaries and helping to establish a fully functional BBB.^[^ ^24^, ^25^, ^26^ ^]^

Tumors that originate in glial cells are called gliomas and they account for more than 70% of all brain tumors.^[^ ^18^ ^]^ The most frequent adult glioma (65%) is GBM, which develops from astrocytes.^[^ ^19^, ^27^, ^28^ ^]^ The WHO classifies GBM as a grade IV astrocytoma, accounting for ≈12–15% of all intracranial neoplasms and 60–75% of astrocytic tumors.^[^ ^18^, ^19^, ^27^ ^]^ Combining the histopathological features of GBM—nuclear atypia, vascular thrombosis, microvascular proliferation, mitotic activity, cellular pleomorphism, and necrosis^[^ ^19^, ^27^ ^]^—with a putative cancer stem‐likecell subpopulations and a plethora of epigenetic and genetic lesions, makes this type of cancer one of the most multifaceted human tumour, and therefore it is incredibly difficult to treat.^[^ ^19^ ^]^

As implied by the moniker “multiforme,” GBM is characterized by a widespread inter (different between tumors) and intra‐tumoral (within the same tumour) heterogeneity.^[^ ^18^, ^19^ ^]^ Heterogeneity can present in multiple different ways within the same tumour including molecular, metabolic, microenvironmental, and vascular heterogeneity. Devastatingly, these factors can vary within the same tumour leading to regional variations in therapy response and cellular behaviors. This makes clinical management and long‐term survival a much greater challenge and is confounded by the lack of treatment options available. Overall, there is extensive intra‐tumoral heterogeneity displayed by GBM and that this occurs at the genetic, metabolic, and microenvironmental levels which we later discuss (see Section 7.1.). Brief examples include: drastic variations in gene expression at spatially distinct regions of the same tumour,^[^ ^29^ ^]^ mosaicism in the expression of various receptor tyrosine kinases (RTKs) leading to multiple mechanisms of furthering their proliferation,^[^ ^30^ ^]^ variation in the vasculature leading to areas of high and low angiogenesis with varying rates of perfusion,^[^ ^31^ ^]^ and alterations to gene expression within the GBM microenvironment, potentially leading to improved capacity for invasion.^[^ ^32^ ^]^

Inevitably, GBM recurs even following gross total resection and the standard of care. This is due to a lack of specificity in the treatments combined with the highly adaptable behavior of GBM cells allowing them to become resistant to treatment. Recurrence has been suggested to occur following the survival of brain tumour initiating cells which are capable of re‐populating tumour mass and heterogeneity.^[^ ^33^ ^]^ Taken together, we show that GBM presents a unique challenge for researchers and clinicians in order to understand how aspects of heterogeneity, BBB, resistance, and recurrence can be targeted via electrotherapy techniques.

## 3 Electrophysiology

The nervous system has recently been implicated to play a critical role in cancer progression for tumors occurring within the CNS. Contrary to long standing belief, non‐neuronal derived cancerous cells including prostate,^[^ ^34^ ^]^ glial and glia‐derived glioma among others retain the expression of voltage‐gated ion channels^[^ ^35^ ^]^ and neurotransmitter receptors.^[^ ^36^ ^]^ While these cells do no exhibit true action potentials,^[^ ^37^ ^]^ they do display oscillations at the membrane potential (*V*_m_) which, when depolarized, are functionally relevant for glia‐mediated neurotransmitter uptake and release^[^ ^38^ ^]^ and proliferation.^[^ ^39^ ^]^ Hence, it is unsurprising that the communication between neurons and cancer cells is a key pathophysiological trait of gliomas.^[^ ^40^ ^]^ Electrophysiological understanding of GBM is paramount for the progression of targeted electrotherapies, therefore, we briefly discuss recent developments of their involvement in glioma.

### 3.1 Ion Channels

Ion channels are responsible for cellular homeostasis and regulate the influx and efflux of ions required for cell metabolism and function. Glioma cells exhibit vast expression of ion channels including sodium (Na^+^), potassium (K^+^), calcium (Ca^2+^), chloride (Cl^−^), and transient receptor potential cation channels. Blockade of a range of ion channel classes has been demonstrated to reduce the viability of glioma stem‐like cells (GSCs) in vitro thus providing more avenues to explore for novel, targeted therapies.^[^ ^41^ ^]^

K^+^ channels are responsible mostly for large efflux of K^+^ from the intra‐cellular space which aids the cell in maintaining a negative resting potential. They are classified as inward rectifying K^+^ channels (K_ir_), Ca^2+^ activated K^+^ channels and voltage‐gated K^+^ channels (K_v_) which form the largest group of ion channels in the membrane. K_v_ channel expression is altered across various cancers and is well known for roles in cell proliferation and neoplastic progression.^[^ ^42^ ^]^ Specific examples of ion channels involved in GBM include human ether‐a‐go‐go related gene (hERG), K_v_3.3, and PIEZO1. hERG, encoded by the KCNH2 gene, is a delayed rectifier K^+^ channel most commonly found in the heart where it is critical to the rapid repolarization of the ventricular action potential. Mutations in or pharmacological blockade of the hERG channel can result in fatal arrhythmias stemming from altered channel function.^[^ ^43^ ^]^ In GBM, increased expression of hERG correlated with worse survival in a tissue microarray whilst patients treated with inhibitors of hERG showed better survival than those who did not. Interestingly, patients receiving multiple hERG blockers showed greater survival than patients just receiving one which indicates that there is merit in increasing the level of hERG inhibition.^[^ ^44^ ^]^ Systemic administration of hERG blockers would present a risk of patients developing potentially fatal arrhythmias, therefore novel drug delivery mechanisms may be of benefit but need to be carefully considered. K_v_3.3, encoded by the KCNC3 gene, is also a delayed rectifier K^+^ channel which has a role in repolarizing rapid fire action potentials in the cerebellum.^[^ ^45^ ^]^ It has been shown that higher channel expression correlates with better patient survival—note this is in opposition to hERG channels indicating that simple K^+^ ion flux across the membrane is not the driving force of malignancy. Studies on differentially methylated regions found that the KCNC3 gene was hypermethylated in GBM samples compared to controls which typically indicates silencing of gene expression.^[^ ^46^ ^]^ Interestingly, another study found that KCNC3 expression was enriched in the GSC compartment but not in the bulk GBM tissue.^[^ ^41^ ^]^ Taken together, this information suggests that overall tumour expression of KCNC3 is low, but that it is specifically enriched in GSCs which are known to be involved in insidious tumour processes such as local GBM metastasis, resistance and recurrence (see Section 7.4.3.). A final example lies in PIEZO1, a mechnosensitive channel that permits the movement of cations across the membrane in response to mechanical forces on the membrane.^[^ ^47^ ^]^ PIEZO1 has been found to have higher expression levels in high grade gliomas (WHO grade III and IV) than in low grades (WHO grade II), further to this the PIEZO1 gene was downregulated in isocitrate dehydrogenase (IDH) mutant gliomas irrespective of grade. This was later found to be due to genetic hypermethylation in IDH mutant gliomas as part of the wider epigenetic glioma CpG island methylator phenotype (G‐CIMP) signature. Further in vitro testing showed that genetic knock‐down of PIEZO1 in GBM cell lines caused a reduction in growth whilst the same experiment conducted in GSCs caused an inhibition in the sphere‐forming capabilities of the cells.^[^ ^48^ ^]^This data indicates that PIEZO1 plays a role in the growth rate of glioma cells. Additionally, it was also shown that high expression levels of PIEZO1 correlated with a much shorter survival time and that this may serve as a novel prognostic marker.^[^ ^49^ ^]^ It should be noted that the positive correlation between high PIEZO1 expression and growth rate may not be strictly causative since the increase in PIEZO1 expression is likely preceded by earlier genomic events such as IDH mutation. However, PIEZO1 still holds an important role in controlling tumour cell growth and has the advantage of being targeted either pharmacologically to reduce ionic flux or mechanically to alter its signaling capability.

Ion channels present a unique opportunity as drug targets, as they are membrane‐bound and therefore easily accessible from the extracellular space. Previous studies have highlighted that targeting specific K^+^ channels with inhibitors can increase susceptibility of GBM cells to TMZ treatment, thus potentially helping to overcome resistance to TMZ.^[^ ^50^ ^]^

Both Cl^−^ and Ca^2+^ have been suggested to facilitate migration behavior of GBM cells. Additionally, oscillatory changes in intracellular Ca^2+^ have been hypothesized to initiate GBM invasion.^[^ ^51^, ^52^ ^]^ Mutations in Na^+^ channels of GBM samples have shown to have shorter survival than mutations in K^+^ or Ca^2+^. In particularly, epithelial Na^+^ channels which fall into amiloride‐sensitive Na^+^ channels are associated with proliferation and invasion in many cancers.^[^ ^53^ ^]^ Glioma ion channels^[^ ^35^ ^]^ and inhibitors^[^ ^39^ ^]^ used as a treatment modality have been reviewed. Combination of channel inhibitors with electrotherapy could lead to greater understanding of the cellular mechanism due to induced electric fields.

Unlike neuronal excitability which can be detected via multiple techniques, the excitability of glioma cells is difficult to detect with high selectivity due to the minute signals they present. Ultra‐sensitive platforms have been developed^[^ ^37^ ^]^ and will be crucial for understanding ion channel regulation thus electrophysiology of glioma.

### 3.2 Neuron‐Glioma Interactions

In high‐grade gliomas, progression is vigorously regulated by neuronal activity (Figure 1). Since neurons are crucial components of glioma microenvironments and regulate activity‐dependant malignant growth, it was suggested that gliomas may also engage in synaptic communication which could be fundamental to its progression.^[^ ^54^ ^]^ Validating this hypothesis, Venkatesh et al.^[^ ^38^ ^]^ show that neuron‐glioma interactions involve electrochemical communication via bona fide *α*‐amino‐3‐hydroxy‐5‐methyl‐4‐isoxazolepropionic acid (AMPA) receptors at the neuron‐glioma synapses. They further show that the interconnections form an electrically coupled network since neuronal activity evoked non‐synaptic activity‐dependent K^+^ currents. Using in vivo optogenetics, the authors showed that depolarized glioma membranes promoted proliferation while pharmacological/genetic blockers of electrochemical signaling inhibited growth. Ultimately, their findings postulate that synaptic and electrical integration into neural circuits promote glioma progression.

As neuronal activity promotes glioma growth, gliomas too increase neuronal activity in preclinical models of GBM.^[^ ^55^, ^56^, ^57^ ^]^ Electrocorticography recordings in awake human subjects with cortical GBM confirm neuronal hyper excitability in the disease‐infiltrated brain compared to adjacent parenchyma.^[^ ^38^ ^]^ Interestingly, the primary excitatory neurotransmitter, glutamate, is implicated in the bidirectional interactions between neurons and glioma cells.^[^ ^58^ ^]^ Thus, increased extracellular glutamate in and around adult gliomas have been reported. It is therefore plausible, that the elevated extracellular glutamate could explain common clinical seizures associated with GBM.^[^ ^55^, ^56^, ^59^, ^60^ ^]^ The take‐away message is that neurons within the tumour microenvironment and glioma cells are reciprocally engaged in a feedback loop as the tumour grows. Thus far, the literature suggests that glioma progression is regulated by electrochemical driving forces from interconnected neurons, however, neuron's role in glioma initiation remains an unanswered question.^[^ ^58^ ^]^

## 4 Pulsed Electric Fields

PEFs are used in several biological, medical, environmental, and food processing applications. Short duration (1–10 µs pulse width), mono‐ or biphasic waveforms have been used to kill pathogens and spoilage microorganisms in vegetal or animal tissue,^[^ ^61^, ^62^ ^]^ as well as, extraction of cellular compounds and growth stimulation in fungi, soy, microalgae, and other cells.^[^ ^63^ ^]^ The biological effects induced by electric fields had been observed and studied since the early 1700s, most notably Jean‐Antione Nollets’ “electric boy” experiment. In recent decades, PEFs have provided a non‐thermal tissue ablation treatment technique for a variety of malignant neoplasms. Broadly, this modality,termed electroporation, can provide both reversible and irreversible effects to biological cells depending on the applied field parameters. The terminology used in this field is typically described by the intended use of electroporation^[^ ^64^ ^]^ which is practiced across multiple modalities as we discuss. For simplicity, in this section we introduce electroporation generally, before describing applications of electroporation,ECT and nsPEFs, and how these are used in glioma treatment. Finally, we introduce IRE protocols and how they have been applied in gliomas to date.

### 4.1 Electroporation and Electropermeabilization

Electroporation variations in cancer treatment are biophysical tissue ablation techniques where rod/needle‐like electrodes are strategically placed directly in or surrounding the lesion of interest. Electroporation can be an invasive modality and therefore limited by surgical intervention. Nonetheless, appropriate placement of one or multiple electrodes which are selectively stimulated with appropriate combinations of field strength and pulse duration and can accordingly interfere with a cells *V*_m_, which sits anywhere from −50 to −90 mV in non‐proliferating glia.^[^ ^65^ ^]^ When the strength of ***E*** is large enough to induce a *V*_m_ ≈ 0.25–0.5 V, the membrane of the cell becomes unstable and nanoscale pores are introduced. Here, the effect is considered reversible while an induced *V*_m_ ≈ 1 V results in irreversible damage. The electroporation phenomenon was termed by Neumann and Rosenheck.^[^ ^9^ ^]^ Mechanisms here are discussed in brief as detailed discussion on distinguishable mechanisms of electroporation and electropermeabilization are already available.^[^ ^66^ ^]^

#### 4.1.1 Mechanisms

**Figure 2:**
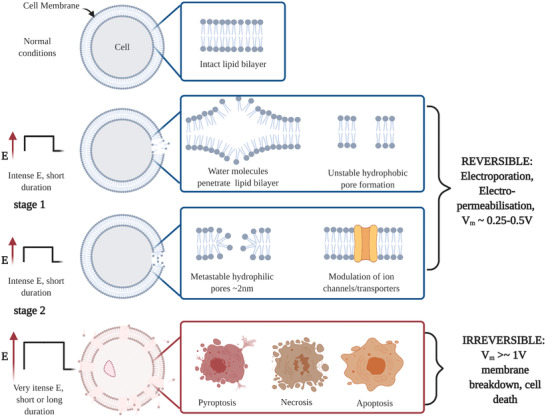
Stages of electroporation. Short but intense electric fields (induced *V*_m_≈0.5 V) force water molecules to penetrate and disrupt the lipid bilayer in the cell membrane leading to unstable hydrophobic pore formation (stage 1). The hydrophilic heads of the lipids begin to reorientate to form a metastable pore of ≈2 nm wide allowing small molecules to enter the cell (stage 2). Depending on the strength and duration of the applied field, modulation of ion channels/transporters have been reported. When the field strength is very intense (*V*_m_ ≥ 1 V), the cell membrane breaks down due to lack of homeostasis and several cell death pathways have been suggested including pyroptosis, necrosis and apoptosis. Created with BioRender.com

The extent of electroporation can be described according to the applied electric field's parameters used to induce changes in *V*_m_. The cell membrane is comprised of a tightly bound lipid bilayer which allows for a semipermeable ion transfer of small molecules between intra‐ and extracellular spaces. Larger molecules are transferred via ion channels or transporters. As illustrated in **Figure** **2**, during low to medium field strength and/or respective long/short duration electroporation (e.g., 8 pulses of ***E*** ≈ 1.3 kV cm^−1^, with pulse width ≈99 µs for reversible ECT^[^ ^67^ ^]^) water molecules begin to penetrate the lipid bilayer inducing unstable hydrophobic pores (stage 1). During stage 2, the lipids re‐orientate their polar head groups causing metastable hydrophilic pores throughout the cell membrane.^[^ ^64^ ^]^ Eventually a state of electropermeabilization is reached, allowing small molecules to traverse the membrane, whose permeability increases due to a range of physical or chemical mechanisms triggered by the applied electric field, such as modulation of membrane protein functions.^[^ ^66^ ^]^ The extent of electroporation and thus of electropermeabilization can be quantitatively estimated and predicted according to changes induced in *V*_m_. When the strength of ***E*** is substantially increased with a relevant pulse width to induce a *V*_m_ ≈ 1 V, the membrane breakdown reaches an irreparable state leading to cell death.

#### 4.1.2 Quantitative Descriptions

Induced changes to a cells *V*_m_ due to an applied electric field were first described mathematically by the Schwan equation which considers a spheroidal cell shape.^[^ ^68^ ^]^ In 1988, Glaser et al.^[^ ^69^ ^]^ described the formation of pores induced by electric breakdown of the lipid bilayer as a function of pore radius and time. Further, in 1999, Neu and Krassowska^[^ ^70^ ^]^ introduced the asymptotic model of membrane electroporation according to the Smoluchowski equation governing the distribution of pores to describe hydrophilic pore formation occurring beyond a critical radius. The required energy to form critical pores which lead to membrane rupture decreases with increasing *V*_m_.^[^ ^71^ ^]^

Using similar approaches today, Kotnik et al.^[^ ^66^ ^]^ describe that even when the *V*_m_ exceeds 450 mV, if the pulse duration is short enough, and *V*_m_ returns to 0 V prior to pores expanding beyond 20 nm, irreversible breakdown can be avoided. Overall, membrane porosity is dynamic and numerical solutions suggest that reversible field strength sub‐microsecond pulses will induce millions of pores at ≈1 nm while longer pulses at similar strengths produce up to tens of thousands of pores in a cell but at a much greater pore radius ≥10 nm.^[^ ^66^, ^72^ ^]^ The prediction of these models strongly correlates with experimental results and this lay the foundation of experimental planning.

**Figure 3:**
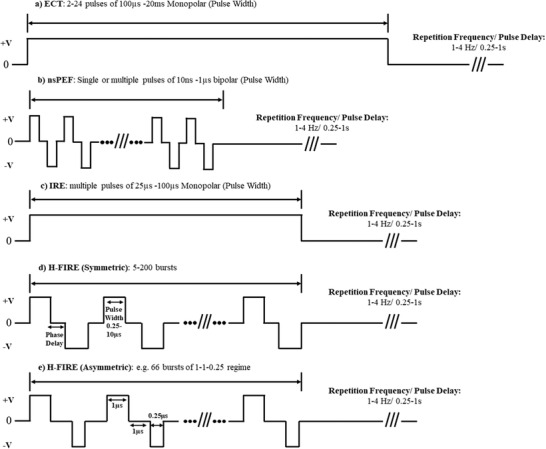
Various electrical regimes used in electroporation techniques. a) ECT are typically monophasic, long in duration (up to 20 ms) with tolerable, reversible field strengths. b) nsPEFs are biphasic and use the shortest duration of all electroporation techniques thus require substantial field strengths to achieve electropermeablization or irreversible effects. However, in this regime, most effects are observed intracellularly. c) IRE traditionally involves a short duration (≈100 µs), monophasic regime at lethal field strengths. d) High‐frequency IRE (H‐FIRE) are second generation IRE waveforms involving biphasic short duration bursts at lethal thresholds to induce cell death. e) H‐FIRE regimes with asymmetric inter‐pulse and inter‐phase delay.

Throughout the literature, there is poor consistency across stimulation protocols which make it difficult to compare results. Previous reports emphasize the effect of electroporation is primarily dominated by two parameters: ***E*** (thus applied voltage and distance between electrodes) and exposure duration (pulse width in nanoseconds to milliseconds), where strength‐duration curves were used as a metric to identify pulsing parameters (U.S. patent no. US8282631B2). However, since different cell types have distinct electrical properties and sizes, these curves should be appropriately calibrated. Additionally, responses have shown to vary with temperature^[^ ^73^ ^]^ and osmotic pressure.^[^ ^74^ ^]^ More generally, the waveform of use is particularly important for the target application as shown in **Figure** **3**. What is not clear in current literature is how the variation of electrode materials, sizes, and shapes effects the field distribution. The resulting current flowing through the electrodes would be a useful parameter to include in future literature, as well as, the electrochemical impedance spectra of the electrodes and effective (potential) electrochemical reactions that may occur for the durations used in experiments much like the expectation in neuromodulation studies. A description of the electrical coupling (direct, capacitive, inductive or a combination) should be included. Further details are provided in Section 8.

### 4.2 Electrochemotherapy

ECT in oncology is an application in which electroporation is used to introduce cytotoxic drugs to malignant cells. Although the specific mechanisms in antitumor effectiveness in ECT treatments are not fully understood, the basic understanding is that the reversible permeabilization of the cell membrane enhances the cytotoxic uptake of chemotherapeutics.^[^ ^75^, ^76^ ^]^ The first demonstration of ECT was performed in 1987,^[^ ^10^ ^]^ and began routine use in 2006.^[^ ^77^ ^]^ ECT has been studied both pre‐clinically and clinically for a range of cutaneous, sub cutaneous and deep‐seated tumors including liver, lung, skin, ovary, prostate, pancreas, colorectal, head and neck, and bone.^[^ ^64^, ^78^ ^]^ In the brain, the challenges arise mainly due to the excitable tissue and intraoperative difficulties. As we mention in our earlier sections, aggressive brain tumors like GBM are heterogeneous and are either inherently or progress to become resistant to molecularly targeted drug therapies. In addition, the BBB which is designed to limit toxic perfusion presents an overwhelming task for poorly permeable cytotoxic drugs to reach the tumour.

ECT presents an alternative mechanism for difficult‐to‐treat tumour cells by making them more permeable and using electrophoretic type driving forces. For clinical ECT, patients are typically administered with an intra‐tumoral or intravenous chemotherapeutic agent that spreads throughout the tumour vasculature.^[^ ^78^ ^]^ Following this, the electrodes are placed in or around the tumour according to the treatment planning software of use (for example, Pulsar coupled with Cliniporator VITAE) and deliver short, defined bursts to electrophoretically drive molecules into the target permeabilized cells. Typical protocols for ECT use between 2 and 24 monopolar pulses of 100 µs–20 ms durations with respective therapeutic field strengths.^[^ ^79^ ^]^ After a short period of time (seconds to minutes depending on the pulse parameters) the membrane begins to reseal and the drug can exert its cytotoxic effects. As mentioned earlier, TMZ is typically the physician's choice chemotherapy for GBM since it is able to penetrate the BBB. However, Bleomycin and Cisplatin are charged drugs currently impermeable to the brain but have demonstrated high therapeutic efficacy in alternate tumour models when used with ECT.^[^ ^80^ ^]^ Thus, permeating the BBB creates many explorative opportunities for drug therapies that are successful in alternate tumour models. To date, the main methods of ECT in the brain target BBB disruption rather than tumour infused electroporation. We discuss the findings of BBB disruption in Section 5.3.

Preclinical validation of intra‐tumoral ECT in rat glioma models resulted in 9 of the 13 rats showing tumour regression (69% complete response rate).^[^ ^16^ ^]^ The ECT protocol in this study was delivered first with 42 IU of bleomycin contained in a 14 µL pump and the electric field was delivered at 4 repetitions of 8 pulses of 100 V, of 100 µs pulse width every 1 Hz. The authors explored the safety of ECT in the brain on healthy rats (tumour free). Subsequent MRI data demonstrated that the treatment area was subjected to necrosis and revealed fluid‐filled cavities highlighting that some risks may include oedema, infection or haemorrhage. An additional consideration would be risk of exciting surrounding tissue prompting epileptic events. While this work provides encouraging evidence for the use of ECT in the brain, future studies must mitigate and overcome these risks by optimizing electrode designs, field distributions, and drug dosages.

Large electric field amplitudes at low frequencies would be expected to cause hydrolysis or electrochemical reactions which create additional complications in the brain. However, Bonakdar et al.^[^ ^81^ ^]^ showcased no chemical reaction or bubbling which they suggest is likely due to the short pulse durations and low currents (100 µs, ≈2 mA). Moreover, intracranial tumour electroporation electrode geometry and performance optimization have been studied,^[^ ^82^ ^]^ and neurosurgical techniques have been reviewed.^[^ ^83^ ^]^

### 4.3 Nanosecond Pulsed Electric Fields

Conventional electroporation‐based techniques involve electric fields delivered in the micro‐ to millisecond range with relative understanding of cell membrane disruption. The sub‐microsecond regime, nsPEF, has shown to induce intracellular effects (i.e., nucleic membrane disruption).^[^ ^84^ ^]^ Although electropermeabilization is induced by nsPEF, the strength and duration of the pulse can lead to nano‐sized pores and electroporation effects.^[^ ^85^, ^86^, ^87^ ^]^ Given these parameters, cell electroporation and permeabilization can be reversible and membrane recovery can be in the range of minutes while intracellular repair may take hours.^[^ ^66^ ^]^ Electropermeabilization has previously been described by Kotnik et al.^[^ ^66^ ^]^ to occur in stages: Initiation, expansion, partial recovery, membrane resealing, and memory.

Cell death mechanisms via sub‐microsecond pulses initially showed to be apoptotic in various cell lines and tumour tissue including through caspase activation,^[^ ^88^ ^]^ evidence of indicated intracellular Ca^2+^ release,^[^ ^89^ ^]^ loss of mitochondrial membrane potential,^[^ ^90^ ^]^ and DNA damage.^[^ ^91^ ^]^ Interestingly, while excess Ca^2+^ is known to cause microtubule instability,^[^ ^92^ ^]^ Carr et al.^[^ ^93^ ^]^ recently demonstrated in human U‐87 GBM cell lines, that nsPEFs affect the microtubule network by a mechanism that is independent of intracellular Ca^2+^ concentrations or osmotic swelling. In addition, nsPEF have shown to effect tumour growth^[^ ^94^ ^]^ and vascular perfusion^[^ ^95^ ^]^ in vivo. For detailed discussion of nsPEF induction of cell death mechanisms we refer the reader to this study by Beebe et al.^[^ ^96^ ^]^

Since the CNS combines both excitable and non‐excitable tissue, the safe use of electroporation‐based protocols in the brain requires a well‐designed approach. Recently, a comparison by Dermol‐Černe et al.^[^ ^97^ ^]^ show for pulses of 10 ns and 10 ms duration, plasma membrane depolarization thresholds in both excitable and non‐excitable cells (including GBM), indicated that excitable cells require greater field strengths to depolarize than non‐excitable cells in vitro. Similarly, depolarization was achieved at lesser field strength by the increasing pulse duration. Quiescent neuronal cells exhibit *V*_m_ ≈ 90 mV as mentioned, proliferating cancer cells display depolarised *V*_m_ compared to their healthy counterparts.^[^ ^65^ ^]^ It is therefore likely that various cancer cells, particularly, those expressing various voltage‐gated ion channels are susceptible to depolarization at lower field intensities and thus appropriate stimulus parameters would allow for selectively addressing them without affecting surrounding healthy neuronal tissue.

Rapid membrane depolarization of neurons was shown by Pakhomov et al.^[^ ^98^ ^]^ to occur within 1 ms of 200‐nsPEF electroporation and that voltage gated Ca^2+^ channels did not contribute to depolarization. However, the opening of voltage gated Na^+^ channels which peak 4–5 ms after nsPEF application could lead to action potential generation. Consistent with other studies, nsPEF amplitudes above 1.5–3 kV cm^−1^ induces electroporation effects and at these thresholds there is risk of evoking action potentials. The suggestion in these cases is that longer duration pulses (100 µs–10 ms) allow time for voltage gated ion channels to respond and ultimately reduces the risk of firing action potentials.

Burke et al.^[^ ^99^ ^]^ recently observed in U‐87 GBM cells that there is a direct interaction between nsPEF (single 10 ns, 34 kV cm^−1^ pulse) and the activation of voltage‐gated ion channels that in turn have downstream effects on non‐voltage dependent channels (see figure 12 in ref. ^[^ ^99^ ^]^). Ion channel modulation studies by nsPEF are ongoing in the field of neurostimulation and cancer treatment. These results suggest that nsPEF protocols below electroporation thresholds present opportunities that may be therapeutically beneficial for cancer treatment and should be considered in future studies.

Ultimately, electropermeabilization will also depend on cell size, cell orientation (with reference to the applied electric field) and cell density.^[^ ^100^, ^101^ ^]^ Comparing the literature, we find lethal thresholds are inconsistent and vary across applications and experiments. This could be attributed to the expression of ion channels, intracellular and extracellular electrical properties, the resting state of *V*_m_ or simply the electroporation protocol used.

### 4.4 Irreversible Electroporation

IRE is a predominately non‐thermal tissue ablation technique that uses lethal field strengths and pulse durations to induce permanent nano sized pores which destabilize cell membranes causing lack of homeostasis and inevitable cell death.^[^ ^12^ ^]^ It was pioneered by Davalos and Rubinsky (U.S. patent no. US8048067B2) in 2003 and is now routinely used to treat various tumors.^[^ ^102^ ^]^ While traditional IRE protocols are relatively safe and demonstrate efficacy for cancer treatment in a variety of organs, the translation to human brain cancer still requires additional research for safety and efficacy. We later discuss the “second generation” protocol, H‐FIRE for treating brain cancers.

#### 4.4.1 First Generation Irreversible Electroporation

There are two generations of IRE. The first‐generation IRE, Nanoknife, was FDA approved for human soft tissue tumors (2008) and commercialized for research purposes in 2009. It includes one or multiple electrode probes that are inserted directly in or near the site of a tumour where ablative pulses are implemented to induce cell death while sparing critical structures.^[^ ^103^, ^104^, ^105^, ^106^ ^]^ IRE traditionally uses short monophasic ***E*** delivered in the target tissue for 25–100 µs pulse width (see Figure 3) at lethal intensities typically greater than 1 kV cm^−1^ to induce a *V*_m_ ≥ 1 V. These fields are typically delivered repetitively every 1–4 Hz, to reach substantial irreversible tissue damage. The mechanisms in which IRE induce cell death is suggested to be apoptotic^[^ ^107^, ^108^, ^109^, ^110^ ^]^ and caused due to the breakdown of the cell membrane. Further, numerical studies show that the size of ablation areas can be estimated to inform adequate treatment planning. Real‐time procedure monitoring can be performed via ultrasound and confirmed ablation with MRI.^[^ ^103^, ^111^ ^]^ IRE has been considered a non‐thermal treatment modality since the cell death mechanism is not primarily caused by tissue heating, yet, that does not exclude it from rising temperatures in tissue. Sano et al.^[^ ^112^ ^]^ show in vitro brain tumour models that for pulse durations on the order of 1 µs, IRE is thermally mediated.

#### 4.4.2 Pre‐Clinical Validation of Irreversible Electroporation

While Nanoknife has been used in various in vivo experiments, few relevant glioma models exist. In 2009, Garcia et al.^[^ ^113^ ^]^ produced the first feasibility results of focal ablation (using Nanoknife) in vivo canine brain tissue using 10 × 50 µs pulses between 500–2 kV cm^−1^ delivered every 4 Hz (a total of 9 times). Although the data was preliminary, this work provided the practicality of non‐thermal tissue ablation in the brain. The complications included oedema, seizures or bleeding (due to needle insertion) all of which have suggested overcoming with corticosteroids and a hyperosmolar agent (oedema) or prophylactic anticonvulsant (seizures). Later, IRE procedures in the brain were further corroborated by Ellis et al.^[^ ^114^ ^]^ in 4 normal canine brains demonstrating that the volume of ablation correlates with the strength of ***E***. The post‐operative histopathologic and ultrastructural assessments of this work detailed by Rossmeisl et al.^[^ ^115^ ^]^ reveal that at 72 h following treatment, cell death is primarily mediated by necrosis. Further, the authors found that no significant activity of caspase‐3 or caspase‐9 was evident, and therefore apoptosis was not a significant contributor to cell death following IRE.

The first demonstration of using IRE treatment for an inoperable, spontaneous malignant intracranial glioma in a canine patient was published in 2011 by Garcia et al.^[^ ^116^ ^]^ Since canine malignant gliomas present similarly to GBM in clinical, biologic, pathologic, molecular, and genetic properties, they are considered an excellent translation for human brain tumour treatment.^[^ ^117^ ^]^ The pre‐ (1.36 cm^3^) and 48 h post‐ (0.35 cm^3^) IRE delivery resulted in an average 74.2% reduction of tumour volumes. This work indicated tolerability and successful safe tumour ablation with adjunctive radiotherapy, anti‐oedema treatment, and anticonvulsants with minimal exacerbating haemorrhage.

Most recently, traditional IRE protocols were further studied by Rossmeisl et al.^[^ ^118^ ^]^ utilizing the Nanoknife procedure in 7 dog glioma models. The mean pre‐treatment tumour size was 1.9 ± 1.4 cm^3^ with dog 2 and 5 presenting with GBM. The results demonstrated that in 6 of the 7 dogs, IRE treatment was achieved without inducing/exacerbating oedema or significant haemorrhage and produced a median survival of 119 days post‐operative treatment.^[^ ^118^ ^]^ While most adverse effects were minimized or in line with typical post‐operative surgery, one dog experienced severe cerebral oedema. The tumour location in this case was close to the periventricular regions much like the common site of occurrence in human glioma and the oedema was a result of excessive field strength in IRE. This emphasizes the consideration of tumour location and potential effects in the pre‐treatment and planning of IRE in the brain.

### 4.5 High Frequency Irreversible Electroporation

H‐FIRE^[^ ^119^ ^]^ (second‐generation IRE) delivers short, fast bursts of substantialstrength, biphasic electric fields into/around the region of interest. These short, microsecond (1–10 µs) pulsing regimes delivered in a series of bursts achieve the same energized “ON” time as a single monopolar 100 µs pulse used in traditional IRE but requires much greater field strengthto achieve the same lesion size.^[^ ^120^, ^121^ ^]^ H‐FIRE was introduced to overcome some of the existing challenges found in IRE. By utilizing the same energized time as IRE, direct comparisons can be made between protocols that are implemented in clinically relevant models. In addition, the waveform regime does not induce muscle contractions^[^ ^122^, ^123^ ^]^ as previously shown in IRE treatments.^[^ ^124^ ^]^ Numerical work by Dermol‐Černe et al.^[^ ^97^ ^]^ have also shown through the Hodgkin‐Huxley model why H‐FIRE may permeabilize but not excite tissue. Finally, the H‐FIRE bursts are typically delivered every 1 s corresponding to the clinical system delivery rates that are synchronized with patient heart rates.^[^ ^125^ ^]^

An example of a symmetrical H‐FIRE waveform would involve a positive phase pulse width of 2 µs, a 2 µs inter‐phase delay, followed by a 2 µs negative pulse repeated until the total average energized (non‐zero) time would equal 100µs (see Figure 3). This can be repeated for a desired number (5–200 times every 1 s) and is represented in the literature as 2‐2‐2 H‐FIRE waveform. In some cases, asymmetric waveforms may be used where the phase durations, inter‐phase delays or inter‐pulse delays vary. While biphasic pulses achieve similar biological effect to IRE, the inter‐pulse delay is described to be an important aspect of the delivery. A phenomenon termed the “cancellation effect” where the opposing polarity phase of a pulse cancels the effect of the first phase,^[^ ^126^ ^]^ has been observed in both nano and microsecond pulse ranges and is not fully understood.^[^ ^120^, ^127^, ^128^, ^129^, ^130^ ^]^ Vinzinitin et al.,^[^ ^126^ ^]^ show that longer inter‐phase and inter‐pulse delay between biphasic pulses resulted in more significant cell death in Chinese hamster ovary cells. However, the cell membrane permeabilization effect was not as obvious. It is possible to consider that longer inter‐pulse and inter‐phase delays are more effective as it would allow for dissipation of the formed electrical double layer at the electrode‐cellular interface. Sano et al.^[^ ^79^ ^]^ also demonstrated that asymmetric pulses reduced the lethal threshold to induce cell death in both U‐87 (human GBM) and MDA‐MB‐231 BR3 (human brain metastasis from breast) cells when compared to equivalent energy symmetric waveforms.

#### 4.5.1 Pre‐Clinical Validation of High Frequency Irreversible Electroporation

The cellular mechanism induced by H‐FIRE is relatively unexplored. First, H‐FIRE induced cell death dynamics were found to result in both immediate and delayed cell death.^[^ ^131^ ^]^ Mercadel et al.^[^ ^110^ ^]^ studied IRE and H‐FIRE protocols in 3D models of adenocarcinoma cell line BxPC‐3 (pancreatic cancer) in collagen I hydrogel‐based scaffolds. Comparing 100 µs monopolar IRE and various H‐FIRE protocols with different pulse length/ inter‐pulse delays (all of which had a total energized time of 100 µs per burst) the authors indicate that cell death dynamics were consistent with accidental cell death (ACD—instantaneous uncontrollable cell death cause by extreme physical, chemical, or mechanical disturbance) and regulated cell death (RCD—cell death as a result of active signaling transduction modules that are modulated pharmacologically or genetically). For H‐FIRE specifically, they showed a reduced fraction of cells undergoing ACD compared to IRE and suggested that H‐FIRE has sufficient control over selecting the ACD or RCD mechanism via the electric field delivery.

A parametric analysis by Sano et al.^[^ ^131^ ^]^ on the field strength versus pulse width duration effects in H‐FIRE with bursts from 0.25 to 50 µs showed an inverse correlation between the pulse‐width and toxicity in vitro. Despite that equal energy quantities were delivered in each burst the authors suggest that this could be due to membrane charging times. In addition, they demonstrate that the delay between positive and negative polarity pulses (phase delay) has negligible effect on *V*_m_ but significantly affects the nuclear envelope potential. Their numerical results indicate that cells of similar size but with higher nucleus‐to‐cytoplasm ratio (NCR) will achieve greater effects on the nuclear envelope potential than those of a smaller NCR. Subsequently, the NCR and cell membrane permittivity will determine the nuclear envelope charging characteristics. Since malignant cells, particularly infiltrative glioma cells, are often expressed with higher NCR,^[^ ^132^ ^]^ these findings suggest an element of selectivity when using H‐FIRE regimes.

IRE procedures have shown preservation of critical structures and major blood vessels in humans which is an advantage of this technique over microwave or radiofrequency ablation methods.^[^ ^12^, ^133^ ^]^ As H‐FIRE protocols are known to produce rapid and reproducible ablations,^[^ ^106^, ^134^ ^]^ Siddiqui et al.^[^ ^135^ ^]^ evaluated how the presence of critical vascular and biliary structures in vivo porcine liver models were affected by H‐FIRE protocols. While histological examination indicated no visible collateral damage to adjacent structures, Hematoxylin and Eosin (H&E) staining revealed that endothelial cell damage/shedding was present particularly in vessels located proximal to the site of the electrode insertion. For translation into the brain, this is an important remark detailing the significance of predictable lesions and preservation of critical tissue.

Tissue heating is another significant parameter for translation into the brain since temperature changes may cause protein denaturation, oedema and seizures. Fesmire et al.^[^ ^136^ ^]^ evaluate the temperature dependence in 3D tumour models (both U‐118 MG human GBM and Panc‐1 pancreatic cancer) comparing Nanoknife IRE (NK‐IRE) and H‐FIRE pulses to aid clinical treatment protocol development. The main observations were that lethal thresholds for NK‐IRE were consistent across the temperature range (2–37 °C) and that the ablation zone increased by 7% across the ranging temperatures. Alternatively, H‐FIRE was strongly dependent on the treatment temperature where an increasing temperature resulted in a decrease in lethal thresholds and increase in ablation volumes.

In brain specific models performed in canines, Garcia et al.^[^ ^201^ ^]^ provides a detailed explanation of the therapeutic planning for IRE and Latouche et al.^[^ ^235^ ^]^ for meningioma treatment using H‐FIRE. In the H‐FIRE case, patient specific plans were developed by MRI tissue segmentation, volumetric meshing and finite element modelling. Each patient's therapeutic procedure was customized with a 3D generated patient and tumour specific output depicting the expected electric field distribution, Joule heating and electrode configuration to be used in treatment (for further details see their Supporting Information).

As detailed in Section 4.4.2., Nanoknife was used to irreversibly electroporate spontaneous gliomas in canine models and adverse effects were observed in 1 canine. The advancement of H‐FIRE protocols has since presented an opportunity to overcome these effects. Latouche et al.^[^ ^137^ ^]^ used H‐FIRE treatment in a follow up experiment to selectively ablate intracranial meningioma in 3 canines. MRI scans confirmed solitary mass lesions greater than 1 cm in diameter with characteristics of meningioma. 6 month follow up revealed canine 1 alive, seizure free, and no evidence of tumour, while canine 2 was alive, it required escalation of anticonvulsants to control seizure activity, and was suspected to have residual or recurrent tumour presenting in MRI 5 months post treatment. Due to recurrent status epilepticus, canine 3 died in 76 days. In this feasibility study, no post‐operative adverse effects attributed to H‐FIRE were observed. Muscle/nerve excitation or cardiac arrhythmia were not evident during treatment corroborating the advantages of H‐FIRE over traditional IRE, particularly in the brain.^[^ ^122^, ^137^ ^]^ This study provided the first evidence of organ and indication specific feasibility of H‐FIRE for brain tumour ablation and presents an exciting translation opportunity in the near future. Further H‐FIRE studies have since been performed in awake standing horses by Byron et al.^[^ ^138^ ^]^ for superficial tumors. In this application, H‐FIRE was delivered at 2 µs, up to 3100 V, to horses that were treated without general anesthesia, neuromuscular blockades or cardiac—impulse synchronization. Tumour volumes evaluated by physical and ultra‐sonographic examination indicated a reduction in the mean, 68.8%, SD ± 31.39; after 2–4 treatments. While sample size and post‐operative follow up where limited in this study, the clinical potential in soft tissue was demonstrated. This work further supports the safety of H‐FIRE translation in the field of cancer treatment.

Continuing evidence supports the use of H‐FIRE as a novel and feasible treatment modality for difficult‐to‐treat cancers. These efforts have led to the first in human trial of H‐FIRE for the treatment of prostate cancer (NCT03838432).^[^ ^139^ ^]^ The success of the canine ablation models due to the considerable efforts in H‐FIRE research provides prospective treatment for brain cancer and in particularly, GBM, where no cure exists. While significant work has led to the first in human trial, there still exists many challenges to successful treatment outcomes. Some of these challenges include: real‐time field monitoring, demonstration of safety in human, examination of protocols invoking seizures, intraoperative difficulties in surgery. Further understanding of therapeutic variations across different GBM subtypes, microenvironmental changes, efficacy on currently resistant or recurrent cell types, immune response, and combination therapy could greatly advance momentum in this field.

## 5 Tumour‐Treating Fields

TTFs is a relatively recent treatment for management of GBM which uses low strength (***E*** ≈ 1–2 V cm^−1^) intermediate frequency (100–500 kHz) AC electric fields by extracranial application. Contrary to the mostly experimental aforementioned electroporation techniques, this is a method that has entered the clinical domain. Clinically, this treatment has been approved by the U.S. FDA under the commercial name Optune (Novocure) for the treatment of both newly diagnosed and recurrent GBM. Its developers and those supportive of the method theorise that TTFs exhibit anti‐tumour effects by targeting dividing tumour cells whilst sparing other cells in the brain that are not undergoing division. It is surprising how little TTFs mechanism of action is explored beyond physical mitotic disruption, especially given what is known about electrophysiology in this field.

### 5.1 Discovery

From the initial introduction of the concept in the publication by Kirson et al. in 2004,^[^ ^15^ ^]^ the research has focused on investigation of the antimitotic effects of TTFs. Mitosis is known to be a process which occurs in precisely choreographed stages (prophase, prometaphase, metaphase, anaphase, and telophase). Aiming to ensure that a single cell divides into two genetically identical daughter cells, the stages of mitosis must be executed with exquisite fidelity. It has been shown that the antimitotic effect is selective to proliferating cells, and has minimal impact on non‐proliferating cells.^[^ ^15^ ^]^ TTFs perturb cells in mitosis resulting in plasma membrane contractions and instability and the formation of plasma membrane blebbing.^[^ ^15^ ^]^ Additionally, TTFs can affect motility and assembly of intracellular macromolecules during metaphase of the cell cycle, which, in homeostasis, those intracellular macromolecules are required for mitotic spindle formation. In consequence, the disruption leads to mitotic catastrophe, chromosomal breakage forming micronuclei, and cell death.^[^ ^15^ ^]^ This is thought to be a consequence of the effect of the field on polar macromolecules like tubulin dimers during polymerization‐depolymerization responsible for assembly and disassembly of microtubules. Although questioned, the force moment acting on tubulin dimers during this process is sufficient to interfere with proper assembly. During the cleavage formation, the electric field distribution is no longer homogeneous creating DEP force on intracellular charged and polar particles drawing them toward the center of the furrow causing cell destruction and suicide.

### 5.2 In Vitro Approaches to Investigating the Mechanisms of Action

**Figure 4:**
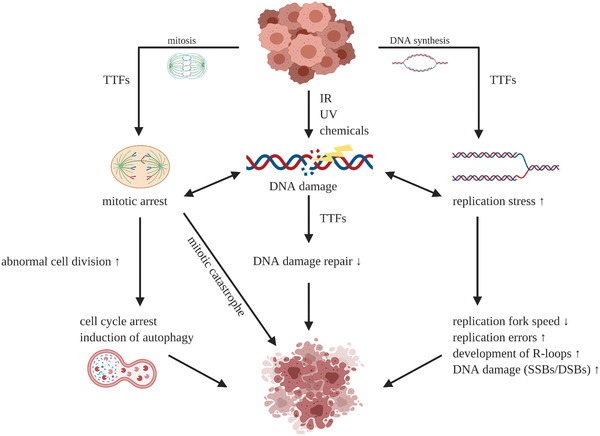
Single cell level mechanism of action of TTFs. TTFs targets mitotic cells inducing, DNA damage, replication stress, and mitotic arrest leading to cell death. Small arrows indicate increase (upward) or decrease (downward). Created with BioRender.com.

A series of in vitro studies reported that TTFs inhibited proliferation and killed tumour cells, including melanoma, glioma, lung, ovary, prostate, and breast cancer cells.^[^ ^14^, ^15^, ^140^ ^]^ These studies observed that the electric field frequencies capable of inhibiting proliferation are dependent of the sizes and shapes of cells.^[^ ^15^ ^]^ Focusing strictly on publications concerning GBM, we notice that most of the research has been conducted using the inovitro system, which was developed by Novocure to aid investigation into mechanisms of action of TTFs, as well as, finding therapeutical combinations adjuvant with TTFs. There are only a few publications describing research where primary GBM cells were used in in vitro setups, with the majority of authors relying on 2D culture of GBM cell lines. Therefore, although these models can provide information regarding general mechanism by which TTFs work, it is difficult to predict the efficacy of the treatment in terms of its impact on various cell types and even tumour subtypes within the tumour mass and tumour microenvironment. Nonetheless, taking together the information from the available publications on the effects of TTFs on GBM cells, we can group and summarize the described mechanisms (**Figure** **4**).

As mentioned, TTFs can disrupt the alignment of several cellular structures, including the spindle structure and contractile ring. Those disruptions often happen during various cell cycle phases—anaphase, telophase, and cytokinesis—and prevent cytoplasm separation. In consequence, this leads to apoptosis. The programmed cell death can be induced via either p53‐dependent or p53‐independent pathway.^[^ ^141^, ^142^ ^]^ Considering that TTFs present the ability to target several cell cycle phases, therapies that disrupt Gap1/synthesis (G1/S) or Gap2/mitosis (G2/M) phases could be combined to enhance treatment. The type of cell death induced upon TTFs treatment may differ between tumour entities and cell lines.^[^ ^140^ ^]^ In U‐87 and U‐373 human GBM cells, TTFs induced autophagy, and suppressed autophagy may attenuate anticancer effects.^[^ ^143^ ^]^ Further, Akt2/mTOR/p70S6K axis are crucial pathways underlying TTF‐induced autophagy.^[^ ^143^ ^]^

Multiple studies have observed the correlation of cell doubling time to TTFs efficacy both experimentally and numerically.^[^ ^140^, ^144^ ^]^ This remark corroborates the results by Giladi et al.,^[^ ^140^ ^]^ which experimentally demonstrates the correlation between TTFs efficacy and cell doubling time, thus for any treatment duration, the efficacy is greater on rapidly dividing cells than quiescent cells.

Interestingly, Neuhaus et al.^[^ ^145^ ^]^ and Li et al.^[^ ^146^ ^]^ have suggested that TTFs have an effect on *V*_m_. Since *V*_m_ of tumour cells are typically depolarised and fluctuate during proliferation,^[^ ^147^ ^]^ TTFs could potentially induce considerable change to *V*_m_ resulting in reduced cell counts. According to Li et al.^[^ ^146^ ^]^ this may explain why healthy glial cells, which feature resting *V*_m_ ≈ −90 mV are unaffected by the relative change in *V*_m_ potentially induced by TTFs. Experimentally, Neuhaus et al.^[^ ^145^ ^]^ demonstrated that TTFs were able to activate Ca_V_1.2 channels in GBM cells. They also demonstrated that clonogenic survival varied across GBM cell lines and that Ca_v_ antagonist may augment the therapeutic effect.

If a tumour cells induced *V*_m_ is depolarised considerably, the consequent changes in ion concentration due to downstream activation of voltage‐gated ion channels and transporters could explain the abnormalities expressed in mitotic cells from TTFs exposure. Conversely, if TTFs exposure induces hyperpolarization, this may explain why cells no longer divide and reduced cell counts are observed since depolarization is believed to initiate mitosis and DNA synthesis.^[^ ^65^ ^]^ Moreover, the fluctuations of *V*_m_ through‐out phases of the cell cycle and mitosis could also explain why different effects are observed. For example, in many cells, depolarisation halts G1/S checkpoint, remains relatively hyperpolarised during S phase, and finally the G2/M transition again exhibits depolarization.^[^ ^65^ ^]^

### 5.3 In Silico Single‐Cell Approaches to Understanding the Mechanism of Tumour‐Treating Fields

Single cells can be modelled using finite element software like COMSOL Multiphysics to investigate potential underlying physics of specific TTFs induced mechanisms. Regardless of whether the model includes a single cell, tumour‐like features or full‐scale head models, the same principle to estimate field distribution is considered. Electric field distributions can be approximated according to a volume conductor model,^[^ ^7^ ^]^ where electro quasi‐static approximations of Maxwell’ electrodynamics equations can be applied and wave propagation terms ignored. This approximation is valid since the wavelength of the applied TTFs frequency in tissue is much larger than the size of the human head. From these assumptions, the electric potential can be computed using Laplace's equation, Equation (1).

(1) $$\nabla \;\times \;\tilde{\sigma \;}\nabla V\;=\;0$$

where $\tilde{\sigma }=\;\sigma +i\omega \varepsilon \;$is the complex conductivity, *ε* is the permittivity and *ω*  =  2*πf* is the angular frequency.^[^ ^142^ ^]^ The boundary conditions typically assume that all interior boundaries have continuity of the normal component of the current density and external boundaries are electrically insulating.^[^ ^148^ ^]^

While TTFs is both field strength and frequency dependant, many studies have also indicated that the direction of the field changes the therapeutic effect for cells that are in telophase.^[^ ^4^ ^]^ Hence, for electric fields that are applied directionally parallel to the cell division orientation (where the furrow is parallel to field direction), the field strength at the furrow is greatest producing maximum DEP force. Wenger et al.^[^ ^142^ ^]^ indicates that the optimal frequency (which they define by when the strength of ***E*** is strongest) is cell cycle stage dependant. For example, for the cell modelled to be in metaphase, the strength of ***E*** is maximum at approximately 10 MHz, however, for a cell modelled in late telophase, maximum ***E*** occurs at ≈100 kHz. This finding suggest that the delivery of TTF should consider sweeping a range of frequencies relative to cell cycle progression. While this is yet to be validated experimentally, a consideration of cell cycle stage dependency should also include the corresponding changes in *V*_m_. Wenger et al.^[^ ^142^ ^]^ also show how variations in cell size will change the exposure to TTFs, in particular, as the cell size increases, the efficacy is reduced.

The mechanism that TTFs interrupts the mitotic spindle formation in early stages of mitosis is said to be unlikely since the forces exerted by the electric field strength are insufficient to cause cytoskeleton disruption.^[^ ^142^, ^146^, ^149^ ^]^ As explained in single cell studies by Tuszynski et al.,^[^ ^149^ ^]^ within the cytoplasm, electrostatic charges would be screened over the distances greater than the Debye length and that the simplified force for unscreened charged according to *F*  =  *qE*, (where ***E*** is static 1 V cm^−1^) would result in 10 or 0.5 pN if Debye screening is accounted for. Similarly, in the work presented by Li et al.^[^ ^146^ ^]^ further show, taking random thermal motions into consideration, the torque and force induced by TTFs at ***E*** ≈ 2 V cm^−1^ are nearly four orders of magnitude smaller than thermal motion energy. This too suggests that the forces are not comparable to that of Brownian motion and would be ineffective to disrupt the tubulin dimer alignment during mitosis.

While direct effects on microtubule polymerization are less likely to be contributing to TTFs induced cell death during prophase/metaphase, telophase and cytokinesis disruption is said to be the main mechanism for cells that progress. The assumptions of homogenous field distributions in tissue vary drastically between electrotherapies making comparisons difficult. Since the electric field distribution within a cell in late cytokinesis is largely inhomogeneous, theoretically, significant DEP forces will develop and cause polarizable particles to move as a result of the induced non‐uniform field acting on their dipole moments.^[^ ^149^ ^]^

Li et al.^[^ ^146^ ^]^ studied DEP forces arguing that previous theoretical studies neglect the inclusion of cytoplasm viscosity and that Stokes drag forces would strongly influence the movement of macromolecules toward the furrow. With this inclusion, the simulation shows at 1 and 10 min time points, no clear indication of particle movement toward the furrow. Since telophase represents a small percentage (2–5%) of the typical mitotic duration (12 h), these results, although not experimentally validated, indicate that macromolecule movement is not the cause of cell death in TTF.

### 5.4 Tumour‐Treating Fields in Clinical Practice

Novocure's Optune system consists of 4 patches containing an array of 9 transducers (36 electrodes in total) that are placed on a shaved head. Each pair of transducer array rests across the left and right, temporal and parietal areas (LR array) and the anterior‐posterior pairs across the supraorbital and occipital regions (AP array). These arrays are positioned optimally according to the treatment planning software, NovaTAL (NovoCure ltd), and deliver biphasic electric fields with a typical 200 kHz center frequency at therapeutic thresholds estimating ***E*** ≥ 1 V cm^−1^ at the tumour location. The commutation time for each electrode pair is 1 s on the left‐right (LR) pair, followed by 1s on the anterior‐posterior (AP) pair repeatedly. The portable device is recommended to be worn for at least 18 h per day for optimal outcomes.

#### 5.4.1 Clinical Trials

The two pivotal clinical trials which had sizable patient populations and focused on outcomes directly related to TTFs were EF‐11 (237 patients) and EF‐14 (695 patients),^[^ ^150^, ^151^, ^152^ ^]^ and the patient registry dataset (PRiDe).^[^ ^153^ ^]^ The EF‐11 clinical trial was designed to investigate TTFs as a monotherapy versus chemotherapy for recurrent GBM patients, and the primary efficacy endpoint was overall survival (OS). The EF‐14 studied TTFs for newly diagnosed GBM patients, and the primary efficacy endpoint was progression‐free survival (PFS). During the EF‐11 trial, chemotherapy was chosen for the control arm due to lack of an established standard of care for recurrent GBM. The primary endpoint, OS, was not superior in the TTFs arm compared to chemotherapy (median 6.6 months vs 6.0 months).^[^ ^150^ ^]^ After receiving FDA approval, the efficacy of TTFs was also assessed in the PRiDe, a large post‐market registry which included all recurrent GBM patients who began TTFs between October 2011 and November 2013.^[^ ^153^ ^]^ The study did not specify the start date for measuring the overall survival from, though the report said that the OS was 9.6 months which in comparison to the 6.6 months reported for the TTFs arm of EF‐11 was increased.

The demonstrated tolerability of TTFs from EF‐11, provided the basis for EF‐14, a phase III randomized clinical trial investigating TTFs with maintenance TMZ versus maintenance TMZ monotherapy.^[^ ^152^ ^]^ In this trial, TTFs plus maintenance TMZ demonstrated significantly prolonged survival compared to TMZ monotherapy—median PFS was 6.7 months versus 4.0 months, and median OS was 20.9 months versus 16.0 months, respectively. Efficacy of TTFs was similar across age, Karnofsky Performance Status, MGMT methylation, extent of resection, or geographic location. Thus, no prognostic indicators were identified for subpopulations of patients that may receive greater benefit from the treatment. Overall, the significant increase in PFS and OS demonstrated in EF‐14 resulted in approval of TTF therapy for newly diagnosed GBM patients.

#### 5.4.2 Computational Head Models

Computational models offer a cheap and efficient way to estimate the electric field distribution and inform optimal transducer placement during treatment planning of TTF. As mentioned, NovaTAL is the proprietary treatment planning software for Optune, and it creates personalized electrode placements based on patient specific data. It was not included as part of the EF‐14 clinical trial but was approved following a clinical system user study.^[^ ^154^, ^155^ ^]^In bespoke systems, properties for the scalp, skull, cerebrospinal fluid (CSF), grey matter (GM), and white matter (WM) can be obtained according to segmentation of MRI (isotropic) or diffuse tensor MRI (anisotropic). In addition to MRI based techniques, water‐content electrical property tomography has also been used for mapping brain tissue conductivities in the intermediate frequency range 200–1000 kHz.^[^ ^156^ ^]^ GM and WM surface meshes (most typically using anisotropic conductivity tensors^[^ ^157^ ^]^) are created in software's like the SimNibs pipeline while other tissue meshes can be modelled using the Brainsuite package. Mimics are an additional software package used to create virtual tumors and to correct bumps or holes in the total mesh. Complete meshes are imported into common finite element analysis packages to solve the electric field distribution . These models are developed to be reflective of the NovaTAL system.^[^ ^148^ ^]^

Recently, Lok et al.^[^ ^158^ ^]^ compared NovaTAL and generated bespoke transducer array positions for cerebellar GBM. Shifting lateral arrays backward and posteroanterior arrays to the lower occipital and upper cervical regions revealed superior field coverage at the tumour location to the NovaTAL‐generated positioning. Similarly, Korshoej et al.^[^ ^159^ ^]^ showed that oblique orientations where the operational electrodes are separated by 45° relative to the sagittal plane, produced greater field strength across tumour locations than standard LP‐AP orientation.

Following the phase 3 EF‐14 trial, Urman et al.^[^ ^160^ ^]^ used computational modelling to correlate the PFS and overall (OS) survival of 119 patients to the relative field distributions. Their models show that when at least 95% (E95) of the combined volume of the gross tumour volume and proximal boundary zone achieved ***E*** ≥ 1.3 V cm^−1^, PFS was 11.9 months and OS was 33 months compared to PFS 7.5 months and OS 21.9 months for ***E*** ≤ 1.3 V cm^−1^. Ultimately, these results suggest that greater ***E*** strength at the tumour location corresponds to improved patient outcomes. For further reviews specific to computational head modelling, we refer the readers to Bomzon et al.^[^ ^161^ ^]^ and Wenger et al.^[^ ^142^ ^]^

Several conclusions can be drawn from computational models, but not without limitations. Most commonly, relating diffuse MRI data to stereotactic in vivo conductivity measurements with specific emphasis on the heterogeneous properties would create more accurate and predictable results. Reducing the time constraint and complexity of creating patient specific models would greatly assist physicians involved in treatment planning and perhaps improve adoption in the clinic.

While TTFs have demonstrated efficacy in vitro, in vivo and in clinical trials, and is an approved treatment protocol for both newly diagnosed and recurrent GBM, there is skepticism and apprehension from both researchers and physicians. Skepticism arises due to incoherent results, with certain clinicians taking a wait it out approach.^[^ ^162^ ^]^ As discussed by Wick's review,^[^ ^163^ ^]^ the main source of skepticism is the lack of a clear mechanism of action in complex models and localized tissue. As discussed in both single cell and more complicated head models, there are contradicting theories which create enough uncertainty to warrant continued research into the mechanisms behind the observed outcomes.

## 6 Intra‐Tumoral Modulation Therapy

Optune as the approved treatment modality for GBM patients has changed the procedures in management of the disease. However, there are various reasons for compliance difficulties for this system. Those are related to operational aspects (e.g., requiring a shaved scalp, dermatological complications, perpetual application) and stigma of using an external treatment system. Treatment efficacy may also be limited by an inability to conform field dimensions to maximize stimulation strength and avoid off‐target injury.

GBM progression most commonly occurs as an extension from the site of the original lesion. The extension has been recognized as aggressive and incessant.^[^ ^164^, ^165^, ^166^ ^]^ Due to this characteristic of GBM, it has been endeavored to design locoregional strategies which could restraint growth of unresectable tumors, thus in consequence preventing recurrences.^[^ ^167^, ^168^, ^169^, ^170^ ^]^ Consequently, there are research groups currently challenging the method of external placement of transducer arrays. If effective, those alternative inventions may prompt development of implantable technology to deliver low‐strength stimulation within tumour‐affected brain regions.

Intra‐tumoral modulation therapy (IMT) has been introduced and tested in vitro and in vivo for the treatment of GBM. The delivery of IMT has been developed in a form of an in‐dwelling device which delivers electric charge to those brain regions which have been affected by the tumour. This method explores how the known electro‐sensitivity of GBM cells can be used as a strategy for treatment in a localized, targeted manner. The device has been designed to allow for sustained and titratable therapy, offering very low maintenance, as well as, hidden away hardware which can have an impact on patients’ quality of life.

The first evidence of IMT was described by Xu et al.^[^ ^171^ ^]^ The study sought proof‐of‐concept evidence for the in vitro anti‐tumour efficacy of pulsed (90 µs pulse width at 130 Hz) small amplitude (*V* = 4 V) electric stimulation continuously delivered to the epicenter of tumour cell preparation via an indwelling electrode. The results showed that the viability of patient‐derived primary GBM cells was reduced under the influence of IMT treatment with negligible impact on primary post‐mitotic rat neurons. Additionally, the study has shown apoptosis and enhanced chemotherapeutic effect in GBM cells treated with IMT. However, it currently is not known whether this treatment shares common mechanisms of action with other electrotherapeutic modalities.

Di Sebastiano et al.^[^ ^172^ ^]^ tested a new profile of parameters, using intermediate frequency at 200 kHz and a sinusoidal waveform to deliver continuously at V ≈ 2 V. Their experiments further confirmed in vitro that primary human and, F98 GBM rat cells but not primary post‐mitotic rat neurons were exquisitely sensitive to low amplitude, sinusoidal pulses at a frequency out of range for neuronal entrainment or thermal injury. Moreover, they presented the use of special purpose, MRI‐compatible bioelectrodes strategically positioned within, or adjacent to, tumour‐affected regions. A 1‐week course of continuous IMT monotherapy produced a significant reduction (19.7 +/− 24.3% with outlier excluded) of mean GBM volume in the living rat brains.

The in vivo test showed that the key feature of IMT is the ability to reach any aspect of the CNS to provide focused, titratable therapy directly within areas of disease. Bioelectrodes could be designed for personalized and comprehensive treatment coverage of GBM resection beds of non‐operated lesions within eloquent or deep‐seated CNS regions. The proximity of the IMT field source to GBM pathology will permit a broad, versatile spectrum of stimulation parameters custom optimized to tumour location and treatment response. Such a concealed, indwelling system is expected to support patient quality of life providing sustained, low maintenance therapy that potently complements radiation, and ongoing chemotherapeutic options.

## 7 Mapping Electrotherapies to Glioblastoma Characteristics

**Figure 5:**
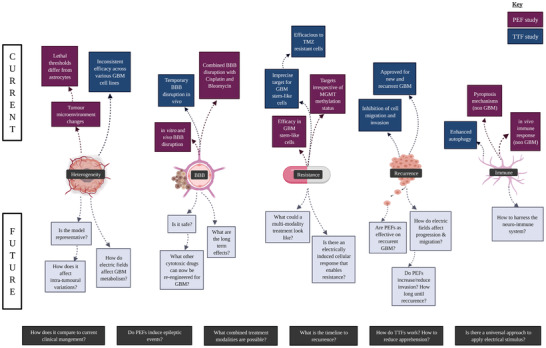
How can electrotherapy be improved in order to better treat GBM? Here we depict both the complex nature of GBM and what strategies of electrotherapy have attempted to overcome these challenges. The prospect of electrotherapies ought to address numerous challenges which are currently poorly understood including various aspects of heterogeneity, BBB, treatment resistance, and recurrence and how innate immunity can be harnessed. Created with BioRender.com

We present in this review thus far that GBM is of complex nature, strongly correlated to neuronal innervations which are subject to reciprocal feedback in glioma growth. We discuss the ways in which PEFs which cause various forms of reversible and IRE have shown pre‐clinical efficacy in gliomas and some alternate tumour models thus far. We describe TTFs in terms of the proposed mechanisms affecting mitotic spindle formation and ductile ring destabilization in GBM and the role of TTF‐induced *V*_m_ changes as a topic of future research. Here, we highlight how each modality of the electrotherapies mentioned show evidence (or lack) of treating or addressing different aspects of GBM intricacy (**Figure** **5**) and questions which remain to be addressed in order to progress the field.

### 7.1 Heterogeneity

#### 7.1.1 Molecular and Cellular Heterogeneity

**Figure 6:**
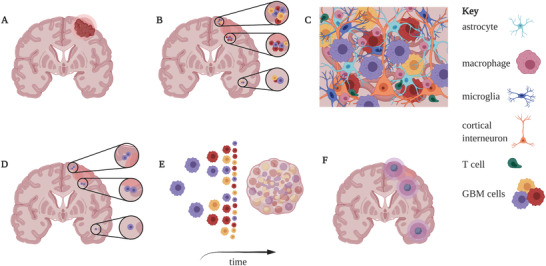
A cartoon representation of the current pathway newly diagnosed GBM patients follow and the most common progression of events (Created with BioRender.com). A) Patient presents to clinic with a brain tumour and receives gross total resection (GTR) surgery. B) Despite GTR and margins, some tumour cells remain close to the resection cavity, and some may have begun spreading further through the parenchyma. C) A representation of the complex heterogeneity of cell types surrounding GBM cells in the brain microenvironment. D) Following GTR, the patient receives TMZ, and radiotherapy which aims to kill remaining sensitive tumour cells; however some resistant cells persist following the cessation of therapies (shown in purple). E) These persisting cells begin the process of re‐populating the tumour and re‐establishing the original heterogeneity. F) Despite standard of care, the tumour recurs either at sites close to or further afield from the original tumour. Recurrences may present on the ipsilateral or contralateral side.

At the molecular level, differences in transcription programmes and DNA methylation levels contribute to overall heterogeneity. The identification of the four transcriptional subtypes; proneural, neural, classical, and mesenchymal, was a ground‐breaking discovery that has great potential to change the classification, diagnosis and treatment of patients in the future.^[^ ^173^ ^]^ The neural subtype has since been shown to likely be an artefact of contamination and has since been discounted as a subtype.^[^ ^174^ ^]^ Different fragments of the same tumour could be classified into different subtypes, with some patients exhibiting up to three different subtypes. Since the different subtypes respond differently to therapies and have differential survival times, the discovery of multiple subtypes existing within the same tumour complicates patient diagnosis and treatment (**Figure** **6**).^[^ ^175^, ^176^ ^]^ Spatially distinct tumour fragments showed that heterogeneity increases with time resulting in different areas of the tumour being classified into different transcriptional subtypes. Further to this, multiple biopsies taken from the same tumour were analyzed for their transcription subtype. The proneural subtype was predominantly found at the leading edge and in regions of invasion whilst the mesenchymal subtype was mostly found in more hypoxic regions such as pseudopalisades or the necrotic core.^[^ ^29^ ^]^ This data showed that not only could more than one subtype be found within the same tumour, but that they occupy spatially distinct regions.

5‐aminolevulinic acid (5‐ALA) is an oral compound used as a tool to aid maximal surgical resection as it produces red fluorescence under blue light selectively within cancer cells. Studies have indicated the fluorescence is not uniform throughout the tumour volume^[^ ^177^ ^]^ and that this spatial heterogeneity of fluorescence intensity could be down to cell‐cell differences in metabolic programmes.^[^ ^178^ ^]^ Spatial heterogeneity has also been indicated through findings demonstrating that the metabolism of tyrosine is higher at the core of the tumour than at its edge. An increase in tyrosine metabolism was found within tumour cells of the MES‐like transcriptional subtype indicating that heterogeneity within cellular metabolism and transcriptional heterogeneity may be linked and contribute to overall poor prognosis.^[^ ^179^ ^]^ Differences in the metabolism of tyrosine in these GBM cell populations may have implications on dopamine and glutamate signaling within the GBM microenvironment. Both neurotransmitters are derived from tyrosine and glioma‐initiating cells have been shown to expressed dopamine receptor 2. Activation of these receptors by dopamine caused a hyperpolarization reminiscent of neurons and long‐term stimulation resulted in a higher sphere‐forming ability in vitro.^[^ ^180^ ^]^ Additionally, tyrosine can be metabolized to produce glutamate, which has been shown to induce glioma cell invasion due to action on glioma cell AMPA receptors across neuron‐glioma cell synapses.^[^ ^181^ ^]^ This research brings into question whether heterogeneously altered tyrosine metabolism in GBM cells has the potential to produce both dopamine and glutamate which can act in an autocrine manner to promote tumorigenesis of surrounding tumour cells. These distinctions are important for electrotherapy since the application of an electric field will not perturb each region with equal strength. Current models using electrotherapy assume homogeneity of the tissue when in fact, understanding the variation in the electrical properties of the different subtypes could provide more realistic data for computational models, thus informing appropriate stimulation parameters and electrode geometries for future bioelectronic devices.

#### 7.1.2 Heterogeneity within the Microenvironment and Immune Cell Population

Glioma cells themselves contribute extensively to the heterogeneity of the disease, however the interaction with non‐tumour cells within the brain as well as the immune system further complicate the heterogeneity. Some of these cell types include; neurons, vascular cells, immune cells, and glial cells.^[^ ^182^, ^183^ ^]^ The role of the tumour microenvironment has raised the possibility of harnessing the immune system to treat gliomas. The role of different types of immune cell in the GBM microenvironment as well as recent advances of immunotherapeutics have been extensively reviewed^[^ ^184^ ^]^ and revealed that alterations in gene expression influence the microenvironment as well as the effect of infiltrating cells on glioma physiology. Briefly, the expression of heparanase in GBM samples following chemoradiation was found to be heterogeneous.^[^ ^32^ ^]^ This is a potential mechanism by which GBM cells can physically alter their environment by breaking down ECM components to aid invasion. Additionally, neuroinflammation may be a driving factor in the acquisition of a more aggressive transcriptional programme and has been demonstrated using exogenous IL*β*1 on proneural GBM cells. These cells slowly gained a more aggressive, mesenchymal transcription programme which shows that the transcriptional programmes of GBM cells are sensitive to input from the microenvironment.^[^ ^185^ ^]^ Concerning the application of PEFs, IRE techniques have shown to induce severe cerebral oedema.^[^ ^118^ ^]^ While this was suggested to be overcome with appropriate pharmaceuticals and electric field parameters, studies should consider the risk of induced inflammation not only from a patient safety perspective, but additionally, for the potential of triggering aggressive transcription and invasion.

#### 7.1.3 High Frequency Irreversible Electroporation in 3D Scaffolds

In 3D cultured GBM microenvironment mimics, Ivey et al.^[^ ^186^ ^]^ investigated if IRE or H‐FIRE protocols could specifically target morphological characteristics of cells in heterogeneous microenvironments. They illustrate that malignant cells (human U‐87 GBM, human DBTRG GBM, rat C6 glioma) had significantly reduce lethal thresholds ***E*** ≈ 530–810 V cm^−1^ compared to healthy counterparts ***E*** ≈ 930–1200 V cm^−1^ (non‐malignant astrocytes, D1TNC1 and PC12 cells). The authors suggest that treatment regimens may leverage these observations by tuning the applied fields between these thresholds such the ablation of tumour cells are achieve while sparing healthy astrocytes. Further, Ivey et al.^[^ ^187^ ^]^ later showed that molecularly enhanced nuclear to cytoplasm ratio with eA1 adjuvant further enhances the selectivity of PEFs to induce cell death in tumors and that overexpressed EphA2 receptors could be exploited in combination treatment regimes.

Cell‐to‐cell interactions in the GBM tumour microenvironment under the influence of PEFs are not well studied and hence not understood. Additionally, specific responses to cellular and molecular variations between GBM samples are yet to be reported in detail. However, there is evidence that PEFs are effective against GSCs as discussed in Section 7.4.4.

#### 7.1.4 Tumour‐Treating Fields and Heterogeneity

Various in vitro studies of GBM cell lines have been used to study the effect and mechanisms of TTFs with some cell lines more responsive than others. The cell size and corresponding dielectric properties will impact the field distribution.^[^ ^142^ ^]^ It is therefore reasonable that the response of electrical stimulus is not unanimous across cell variations. On this notion, TTFs do not show the same therapeutic efficacy across different GBM cells lines. For example, Neuhaus et al.^[^ ^188^ ^]^ showed that U251 and T98G (both malignant human GBM derived) stimulated by TTF combined with Ca^2+^ channel blocker benidipine increased cell death effects in T98G which was not observed in U251 despite that Ca^2+^ entry could be completely blocked in both cells with benidipine. Contrastingly, TTFs decreased aneuploidy and increased clonogenic survival in U251 compared to T98G illustrating selective differences to TTFs. Thus, TTFs alone (U‐251), or in combination with benidipine (T98G) where shown to potentially trigger intrinsic apoptosis.^[^ ^188^ ^]^

For applications in human, patients presenting with GBMs differed in location and size, thus were at different stages of disease. TTFs have exhibited positive outcomes in clinical trials were patients displayed varying heterogeneous characteristics between samples. While interpretation of these remarks are to be taken with caution, the study key endpoints of the EF‐14 trial comparing TTF combination with TMZ (6.7 months OS) and TMZ monotherapy (4 months OS) suggest that TTFs have therpeutic effect for heterogeneous tumours.^[^ ^152^ ^]^

### 7.2 Immune Response

#### 7.2.1 The Effect of Immune Cell Infiltration on Gliomas

Cells of the immune system including microglia and macrophages also show heterogeneity in GBM tissue and their involvement in GBM has been recently reviewed.^[^ ^189^ ^]^ Dysfunctional T cells are involved in GBM and exhaustion of these T cells is due to long term overstimulation. The co‐expression of multiple immune checkpoints is a brain specific T cell signature with triple positive T cells being less able to produce cytokines representing hypo‐responsiveness and T cell exhaustion.^[^ ^190^ ^]^ This renders them ineffective at targeting tumor cells and makes immunotherapies less effective. Thus, methods or strategies that favor a “re‐programming” of T cells into non‐exhausted states prior to immunotherapy may be of therapeutic interest. Studies have found that the mesenchymal subtype has the highest levels of T cell and macrophage infiltration among the different subtypes.^[^ ^191^ ^]^ Given that there is a variation in GBM subtypes within a single tumour, a high level of intra‐tumoral heterogeneity in immune signatures is therefore expected. This indicates that some transcriptional subtypes may be more immunogenic and therefore respond differently to immunotherapies. This also proves a potential need for clinical trials to classify patients based on their majority subtype, as the more immunologically “cold” tumours would respond less and therefore confound trial results.^[^ ^192^ ^]^ Primary GBM samples can be clustered into 2 infiltration profiles,^[^ ^193^ ^]^ one with a significantly increased‐ and another with much lower expression of genes relating to immune suppression compared to normal brain. Overall, data indicates the importance of using immune cell infiltration data when stratifying patients for therapies as patients belonging to the first profile could be better candidates for immune based therapies.

With respect to electrotherapies, neither PEFs’ or TTFs have shown evidence of harnessing or addressing an immune response in GBM models. There is however evidence that IRE protocols in alternate models induced T cell activation and enhanced immune memory.^[^ ^194^ ^]^ Additionally, sub‐cutaneous tumours in immunocompetent mice models showed substantial response to IRE treatment than immunodeficient mice.^[^ ^195^ ^]^ H‐FIRE regimes in breast cancer models show up‐regulation of genes/pathways related to necrosis and pyroptosis (lytic programmed cell death) in vitro.^[^ ^196^ ^]^ More conclusively, gene expression profiling data indicated down‐regulation of genes associated with immuno‐suppression and pro‐inflammatory genes were increased after H‐FIRE treatment. These results suggest that PEFs treatment has the potential to induce an immune response.

Similarly, mechanisms of immunogenic engagements have been considered for TTFs outside the CNS.^[^ ^197^ ^]^ TTFs can interfere with DNA replication fork by repositioning fragments of DNA created during replication, which leads to severe disruption of DNA damage repair and breast cancer 1‐mediated homologous recombination pathways.^[^ ^198^, ^199^, ^200^ ^]^ Moreover, TTFs may cause endoplasmic reticulum stress which triggers adenosine monophosphate‐activated protein kinase (AMPK)‐dependent autophagy^[^ ^201^ ^]^ and/or immunogenic cell death.^[^ ^197^ ^]^ Immunogenic cell death can additionally be initiated by cytoplasmic double‐stranded DNA which leads to activation of pyroptosis via the STING pathway.^[^ ^202^ ^]^ Again, these studies imply that TTFs can induce programmed immunogenic cell death when applied to tissue outside of the brain.

While these results are promising, the microenvironments of these models and their interactions with the immune system diverge from what could be expected in the brain. Consequently, conclusions cannot be drawn from this research without limitation.

Macrophages have become a recent topic of therapeutic interest given that their polarization (M1/M2) is involved in both pathological conditions (including tissue repair, cancer, allergy, and chronic inflammation) and various physiological conditions.^[^ ^203^ ^]^ The complexity of the tumour microenvironment demonstrates the ability of neoplastic brain cells to strongly influence the polarization of tumour associated macrophages.^[^ ^204^ ^]^ Since the membrane potential has been recognized as a key regulator in many biological transitions/functions, macrophage polarization was explored through bioelectric targeting of ATP sensitive potassium channels (K_ATP_).^[^ ^205^ ^]^ The outcome of these experiments suggests that *V*_m_ acts as an instructive signal for macrophage polarization and that K_ATP_ channels are potential targets for immunomodulation. This is an important consideration for uncovering the role of macrophages in electrotherapy and whether bioelectric modulation can be tuned to induce macrophage polarization toward tumour‐killing phenotype. Currently, the role of the immune system in response to electrotherapies in GBM remains an open question.

### 7.3 Blood‐Brain Barrier

Due to the BBB presence, systemic drug delivery to brain tumours remains problematic. BBB acts as a protection for neural tissues by hindering the inflow of potentially harmful compounds from the blood to the brain. Thus, it shields the brain pathogens. Additionally, its function involves regulating ions and nutrients entering and exiting the brain.^[^ ^206^ ^]^

Since the connection formed between endothelial cells lining cerebral blood vessel—adherent and endothelial junctions—regulate trans‐ and paracellular transport across the BBB, those cells are the core anatomical unit of the BBB.^[^ ^207^ ^]^ Research has shown that in human GBM, the connections between endothelial cells are less stable, therefore creating leaky micro‐vessels.^[^ ^208^, ^209^, ^210^ ^]^ Those openings between endothelial cells can result in cerebral oedema.^[^ ^211^ ^]^ Interestingly, multiple groups have observed that the BBB within high‐grade gliomas are disrupted at the center of the tumour mass but intact at the peripheries, thus impeding drug delivery.^[^ ^211^, ^212^, ^213^ ^]^

Electrotherapies present a unique therapeutic advantage to overcome BBB since their modalities are typically physical. ECT aside, electroporation techniques, and IMT protrude the tumour environment, and TTFs are applied extra‐cranially (though they rely on the therapeutic combination of TMZ for maximum efficacy). Hence, the electrotherapy discussion in this section describes how these techniques have permeated BBB.

#### 7.3.1 Electrochemotherapy—The Vascular Lock Effect

**Figure 7:**
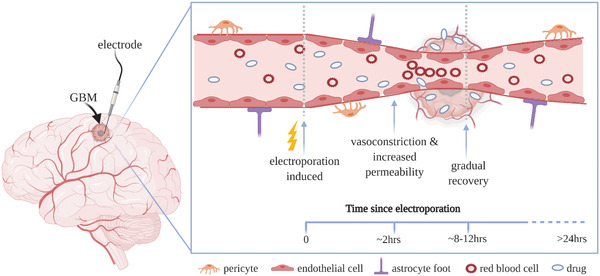
The “vascular lock effect” in reversible conditions. Electroporation induces vasoconstriction, reduce blood flow, and structural changes to tight endothelial cell junctions between 0 and ≈2 h after application. The gaps allow paracellular permeability of ions (i.e., cytotoxic agents) to reach the tumour and extracellular space. ≈8–12 h post application, the vessel is able to gradually recover its shape and function and full recovery is typically evident within 24 h. Created with BioRender.com.

The application of high‐voltage PEF's in tissue induces an immediate constriction but transient reduction in blood flow, a terminology in the field called the “vascular lock” effect (**Figure** **7**). In ECT, this reduced blood flow can be advantageous since it is less likely the combined drug will washout^[^ ^64^ ^]^ or that invasive electrodes will cause bleeding.^[^ ^214^ ^]^

Using intravital multiphoton microscopy techniques, Markelc et al.^[^ ^215^ ^]^ demonstrated in vivo that the main mechanism responsible for increased electroporation induced permeability on normal blood vessels is due to the alterations in endothelial cell‐to‐cell junctions. In addition, the authors elucidate that this may not be the same case throughout the tumour vasculature. Since leaky tumour vasculature and improper organization of endothelial cell‐to‐cell junctions are inherent hallmarks, electroporation effects on their organization may be less profound.

#### 7.3.2 Electroporation‐Induced Blood‐Brain Barrier Disruption for Increased Drug Transport

While several techniques exist to target the BBB temporarily permeabilizing it (ultrasound, osmotic disruption, drug delivery vehicles), many are not able to reach the necessary concentrations in the target tumour tissue.^[^ ^81^ ^]^ Localized reversible electroporation has been used to permeate the BBB to allow systemic cytotoxic drugs to cross and enter the tumour environment in vivo.

Recently, Sharabi et al.^[^ ^216^ ^]^ were able to demonstrate in rat gliomas models that under specific treatment conditions, the BBB could be reversibly disrupted. The experiment included two therapeutics, Cisplatin, which typically has limited perfusion across the BBB but has demonstrated high cytotoxicity in other tumours, and Methotrexate (MTX), which can partially penetrate BBB, is not considered highly toxic and is used in treatment of CNS metastases. The average pre‐treatment tumour volumes were 22.8, 27.6, and 25.2 mm^3^ for control, MTX and electroporation + MTX groups and 17.96 and 24.6 mm^3^ for the Cisplatin and electroporation + Cisplatin groups. It was found that while electroporation induced BBB disruption in combination with MTX was able to slow tumour growth rates (1.02 mm^3^) compared to control group (5.2 mm^3^), there was no significant difference to rats treated with MTX only group (1.7 mm^3^). However, the combination of electroporation and Cisplatin was able to slow the growth rates (0.98 and 1.2 mm^3^ for 90 and 180 pulses) compared to 6.4 mm^3^ in the control group, while Cisplatin delivery alone showed no significant effect on tumour growth rates (3.8 mm^3^). This evidence supports that the combination of electroporation + Cisplatin has the greatest therapeutic benefit. However, these results are to be taken with caution since large portions of this group suffered immediate weight loss, porphyrin discharge around eyes and nose and some rats showing transient partial paralysis of the lower limbs.^[^ ^216^ ^]^

Using voltages from 250 to 650 V, with 4 Hz bursts of 50–90 pulses of 50–70 µs widths a correlation was found in Hjouj et al.^[^ ^217^ ^]^ between the applied voltage and tissue damage and similarly the corresponding volume of BBB disruption. Since the effects of reversible and IRE are transient, the authors also show that BBB disruption was strongly correlated with delayed tissue damage as found by later contrast enhanced T1W MRI data. In similar work, diffusion‐weighted MRI was considered by Mahmood et al.^[^ ^218^ ^]^ as a quantitative method of assessing the electroporation induced membrane permeabilization of rat brain tissue. They found that transient and permanent membrane permeabilization could be distinguished as early as 5 min and 24 h post electroporation treatment. In addition to in situ treatment monitoring, statistical models based on the Peleg‐Fermi model previously used to predict cell death in IRE have also been adapted by Sharabi et al.^[^ ^219^ ^]^ to show predictable BBB disruption.

While animal studies are typically more translatable, in vitro models give less complicated exploration freedom to researchers and still provided quantitative data.^[^ ^220^ ^]^ For BBB modelling to study electropermeabilization, several platforms have been recently employed such as transwells,^[^ ^221^, ^222^, ^223^ ^]^ and microfluidics.^[^ ^81^, ^224^ ^]^

Microfluidic models present alternative in vitro platforms to study and mimic BBB. In the work by Mohammad et al.,^[^ ^81^ ^]^ the reversible and IRE thresholds were investigated focusing on both magnitude and number of pulses using an in vitro model incorporating a “brain endothelial cells on chip” setup of microfluidics to simulate BBB. The experiments conclude that the majority of cells electroporated with 10 pulses were able to recover while 30 and 90 pulses induced irreversible damage for ***E*** ≈646–714 V cm^−1^. The least cell death was observed for ***E*** <400 V cm^−1^. Furthermore, Bonakdar et al.^[^ ^224^ ^]^ present a microfluidic model that enables real‐time measurements of BBB permeability in pre‐ and post‐ treatment conditions of PEFs. Using human cerebral micro‐capillary endothelial cells cultured on a permeable membrane, they suggest that sub‐electroporation pulsed BBB permeabilization can occur via para‐cellular pathways due to cellular deformation and opening of the endothelial tight junctions.

Similarly, Sharabi et al.^[^ ^223^ ^]^ showed transient BBB disruption utilizing 10 pulses of *V*≈ 5–100 V in low voltage experiments and *V*≈ 200–2000 V in high voltage experiments. The durations in each case were 50 µs applied at a frequency of 1 Hz. In the cell viability assay, the results demonstrated that for amplitudes <100 V there was no statistically significant differences when compared to control wells suggesting reversible damage only. The experiments indicated that BBB disruption could not be attributed to electroporation for PEF's <100 V. For amplitudes >1400 V, cell viability was reduced indicating cell death and lethal thresholds.

#### 7.3.3 Tumour‐Treating Fields Targeting Blood‐Brain Barrier

In vitro studies using GBM cell lines have shown that TTFs can increase the plasma membrane's permeability by causing mis‐localisation of specific tight junction proteins (claudin‐5 and ZO‐1).^[^ ^225^ ^]^ Images of the human GBM cell line U‐87and murine astrocytoma cell line KR158B treated with TTFs for 1 h revealed many perforations scattered throughout the plasma membrane.^[^ ^226^ ^]^ The results showed that the perforations allowed the uptake of 20 kDa fluorescently labelled dextran particles. Additionally, in vivo experiments performed in the same study revealed that TTFs could induce BBB disruption for 48–96 h.^[^ ^226^ ^]^ Thus, TTFs application could enhance pharmacological compounds’ delivery to the brain. Moreover, it could be used to increase the uptake of intraoperative agents, for instance, 5‐ALA, to improve intraoperative marking of brain tumour margins.^[^ ^227^ ^]^ Finally, as TTFs can also weaken the mechanism of angiogenesis, there is an opportunity to use TTFs combined with anti‐angiogenic agents like bevacizumab.^[^ ^228^, ^229^ ^]^

### 7.4 Resistance and Recurrence

#### 7.4.1 Recurrence

The average life expectancy of a patient with GBM lies between several weeks and several months after postoperative radiotherapy, with a protracted course when treated with TMZ in addition to radiotherapy alone. Median survival is generally less than one year from the time of diagnosis and most patients die within two years, with most long‐term survivors being given the wrong histological diagnosis at first.^[^ ^1^, ^230^ ^]^ Unless the neoplasm has developed from a lower grade astrocytoma, in more than 50% of cases the clinical history in less than 3 months. Although infiltrative spread is a common feature of all diffuse astrocytic tumours, GBM is particularly notorious for its rapid invasion of the neighboring brain structures.^[^ ^19^ ^]^ Invading cells reside outside the contrast‐enhancing rim of the tumour thereby escaping surgical resection and evading radiotherapy. Thus, recurrence is typically inevitable. However, the generation of metastases outside of the CNS remains very rare since the subarachnoid space and CSF tend to remain unaffected.^[^ ^27^ ^]^ PEF studies are yet to include long term in vivo data that determine the rate of recurrence following electrical intervention. Additionally, excluding ECT, combination treatment with pharmaceutical strategies are yet to be compared within current in vivo or in vitro models. In the current pre‐clinical stage, there is no way to compare the techniques to the current standard of care. This data is crucial to inform the clinical translation roadmap for future interventions.

#### 7.4.2 Patterns of DNA Methylation Show Intra‐Tumoral Variation

Methylation of gene promoters is an epigenetic mechanism for regulating the expression of that gene, thus unwarranted changes to DNA methylation have the potential to severely disrupt gene expression. Low grade gliomas (LGGs) and secondary GBMs are excellent examples of this seeing as they commonly harbor mutations in the IDH1 gene which leads to a widespread hypermethylated phenotype termed G‐CIMP.^[^ ^231^ ^]^ A recent study of LGGs (WHO grade II and III) was able to identify six methylation subtypes M1–M6 which they were able to categorize based on IDH status and tumour type.^[^ ^232^ ^]^ Mutant IDH1 is a precursor for global methylation changes in gliomas, however WT IDH1 tumours also show methylation changes. WT IDH1 primary GBM shows significant inter‐tumoral heterogeneity in the pattern of DNA methylation. Methylation status was accurately correlated with the three transcriptional subtypes, suggesting that DNA methylation is also heterogeneous within GBMs. Patients with shorter time periods between primary and recurrent tumours had greater differences in their promoter methylation which indicates that aggressive tumours are capable of changing methylation status more quickly.^[^ ^233^ ^]^ Spatially separate biopsies from GBM patients showed that different subclasses of methylation status existed within the same tumour. Analysis of the CpG islands showed that samples from within the same tumour, instead of clustering together, had a high degree of cross‐over with samples from other patients.^[^ ^234^ ^]^ This suggests a high level of both inter‐ and intra‐tumoral heterogeneity at the DNA methylation level.

Understanding epigenetic mechanisms and their role in GBM initiation and progression is essential as it is so tightly linked with gene expression and as has been discussed, can be correlated with the transcriptional subtypes of GBM. Being able to pharmacologically manipulate the epigenetics and transcriptomic subtype of GBM would provide routes to systematically eliminate biologically distinct regions of the tumour. Electrotherapies such as ECT would allow pharmacological agents access to their intracellular targets and ECT has the benefit of being bespoke to the location and size of a targetable area. For example, drugs targeting the tyrosine metabolism pathway would be more effective in regions of differential tyrosine metabolism, which corresponds to the visual information provided by 5‐ALA (discussed previously in Section 7.1.1.). Thus, surgeons would be able to visually inspect and identify a candidate region and utilize ECT methods to directly apply the drug of choice.

#### 7.4.3 Treatment Resistance and Glioma Stem‐like Cells

Metabolic phenotypes existing within patient‐derived GSC lines of GBM can be categorized into fast cycling cells (FCCs), and slow cycling cells (SCCs). SCCs generated highly invasive tumours in mouse models extending long processes out into healthy brain tissue whilst FCCs formed more contained tumours with less invasive behavior. SCCs accumulate fatty acids as lipid droplets within cells and switch to lipid metabolism upon glucose restriction or pharmacological inhibition of glycolysis. This suggests that the SCC population of GBM has the capacity to switch energy sources depending on external conditions. SCCs were demonstrated to be inherently more TMZ resistant that FCCs. This combined with their ability to switch to fatty acid oxidation means they are likely the surviving population of standard of care procedures and repopulate the tumour over time.^[^ ^235^ ^]^ This also shows that metabolic plasticity is an ability possessed by only certain cell populations in GBM.

GSCs express unique surface markers, are capable of modulating characteristic signaling pathways which promote tumorigenesis, play essential roles in vascular formation, are less proliferative and often confer treatment resistance.^[^ ^236^, ^237^ ^]^ Further, GSCs contribute to both recurrence and resistance by alteration of DNA damage response pathways,^[^ ^238^ ^]^ creating hypoxic microenvironments^[^ ^239^ ^]^ and through a number of signaling pathways (Notch, RTK and sonic hedgehog).^[^ ^236^ ^]^ While several studies have targeted the stemness signaling of GSC, some studies have looked at inducing differentiation in GSCs via bone morphogenetic proteins signaling activation which markedly attenuated the frequency of GSC sphere formation in vivo.^[^ ^240^ ^]^ Pulsed‐current electrical stimulation has also shown to include differentiation in cultured neural stem cells impacting cell fate and neurite extension.^[^ ^241^ ^]^ This raises opportunity for functional and therapeutic targeting of GSCs pharmacologically, electrically or in combination.

GSCs have been shown to possess an incredibly dynamic ability to adapt to the environmental changes and re‐establish heterogeneity following treatment cessation. This population seems to not be enriched by TMZ, indicating that selection of pre‐existing resistant clones is not a mechanism of resistance.^[^ ^242^ ^]^ This suggests that therapies targeting membrane epitopes of GSCs should be avoided as these cells are able to very quickly adapt their membrane protein expression. A study of the metabolism and efflux of drug therapies showed there was an increase in 23 genes involved in drug metabolism^[^ ^243^ ^]^ which suggests GBM can gain resistance via upregulation of drug metabolism and efflux pathways.

Resistance to TMZ can be acquired via changes to the methylation status of the MGMT gene,^[^ ^3^ ^]^ however alternative mechanisms of TMZ resistance have been recently reviewed.^[^ ^244^ ^]^ Other studies have shown that GBM cells exhibit a heterogeneous expression of MGMT at the cellular level^[^ ^245^ ^]^ and that TMZ‐resistant cells are capable of detoxifying mitochondrial ROS thereby enhancing their survival following TMZ treatment.^[^ ^246^ ^]^ In vitro studies have shown that a loss of inhibition of the JAK‐STAT pathway leads to cells with increased radio‐resistance^[^ ^247^ ^]^ indicating that mechanisms of reinstating this inhibition may be viable treatment avenues. Increased levels of the cytokine CXCL1 in GBM samples have also been linked to radio‐resistance via increased NF‐*κ*B signaling.^[^ ^248^ ^]^ Taken together, these studies serve to show that GBM cells possess a unique ability to endure and survive current SOC procedures.

While physical modalities allow an element of selectivity to resistant cells, combination treatments are typically required for significant therapeutic benefit. Both TTFs and PEFs have demonstrated that they can be efficacious to previously identified resistant GBM cells and GSCs. TTFs combined modality have been presented and compared extensively in vitro and in human while PEFs are still pre‐clinical and require extensive research for clinical translation. The current state of research is however, encouraging.

#### 7.4.4 High Frequency Irreversible Electroporation Targeting Tumour‐Initiating and Glioma Stem‐like Cells

Structural characterization of GSCs have shown evidence of increase nucleus atypia,^[^ ^249^ ^]^ hence, Ivey et al.^[^ ^250^ ^]^ hypothesize that GSCs will be highly susceptible to H‐FIRE over healthy astrocytes with regular nuclear size. They study GSCs in 3D type 1 collagen hydrogel scaffolds and first show that the nuclear sizes of GSCs (GBM10, VTC‐061, and VTC‐064) and U‐251 differentiated GBM cells are significantly larger than non‐malignant healthy astrocytes. Using a 0.5‐2‐0.5 H‐FIRE protocol, the authors demonstrate ablation areas are also significantly greater in GSCs and U‐251 cells when compared to healthy astrocytes and non‐malignant neural stem cells. A similar trend was observed using hyaluronic acid scaffolds which have shown to have more realistic in vivo biological and clinical behavior of the GBM microenvironment.^[^ ^251^ ^]^ The ability for H‐FIRE to selectively ablate GSC that previously were resistant to chemotherapy presents deeply encouraging potential for future treatment modalities.

#### 7.4.5 Tumour‐Treating Fields for Resistant and Recurrent Glioblastoma

Since the anti‐tumour activity of TTF is correlated to cell size, which impacts the optimal frequency of use, GSCs may be less responsive than other tumour cells since their sizes are more heterogeneous and proliferate at a slow rate.^[^ ^237^ ^]^ Cells treated with TTFs have shown to have an increase in both cell volume and granularity^[^ ^141^ ^]^ which have been typically associated with senescence^[^ ^252^ ^]^ and autophagy.^[^ ^253^ ^]^ Since senescence has not yet been observed in TTFs, Shteingauz et al.^[^ ^201^ ^]^ hypothesize that granularity may be associated with autophagosome vesicle accumulation and show that TTFs upregulate proteotoxic stress response triggering activation of AMPK and increased autophagic flux in treated cells. Since autophagy facilitates resistance of tumour cells to antineoplastic agents,^[^ ^254^ ^]^ these findings suggest that greater autophagy functions as a resistant mechanism to TTFs, which could be evaded by targeting autophagy.^[^ ^201^ ^]^

TTFs have been shown in vitro to inhibit cell migration and invasion by inducing a more adhesive cell phenotype. This is achieved through dysregulation of cytoskeletal structures and proteins related to the epithelial‐mesenchymal transition (e.g., actins, vimentin, and cadherin), potentially reducing the likelihood of recurrence.^[^ ^255^, ^256^ ^]^ Clinically, analysis of the TTF phase 3 trial (EF‐15) show that 15% of patients with recurrent GBM responded with complete or partial radiological response.^[^ ^257^ ^]^ In comparison, only 9.6% of patients who received TMZ only exhibited objective radiological response. Interestingly, the analysis also indicated that 44% of GBM tumours in this trial initially display growth before reversing and shrinking 2–7 months later.^[^ ^257^ ^]^ This suggests that TTFs provide a better radiological response.^[^ ^225^ ^]^

Smaller group studies on TTF with combined treatment strategies have been shown. Lu et al.^[^ ^258^ ^]^ compared TTFs with a triple‐drug regimen of TMZ, bevacizumab, and irinotecan versus TTFs with bevacizumab‐based chemotherapies for recurrent GBM patients. While the former group had significantly prolonged median PFS compared to the latter from time of recurrence (10.7 months vs 4.7 months, respectively), there was no significant difference in OS from time of recurrence. Finally, Ansstas et al.^[^ ^259^ ^]^ reported a small case series of 8 patients who received TTFs for recurrent GBM refractory to bevacizumab, of which 5 were subsequently re‐challenged with bevacizumab after progression on TTF. Median OS was 216 days (7.2 months) following initiation of TTFs. Since these studies were primarily focused on subgroup analyses or combination therapies and had much smaller samples sizes than the studies previously mentioned, they add limited insight into the effects of TTFs.

### 7.5 Summary of Known Electrotherapy Risks

The application of electric fields in biological tissue carries inherent risks some of which can be mitigated, reduced or suitably maintained. MRI data has indicated that treatment areas subject to intra‐tumoral ECT showed necrosis and fluid‐filled cavities suggesting oedema, infection, or haemorrhage all of which should be approached with caution and better understood for clinical translation.^[^ ^16^ ^]^ Where electroporation was used to induce BBB disruption in vivo, there were no signs of distress, neurological defects one day post‐treatment.^[^ ^216^ ^]^ However, H&E results revealed haemorrhages along electrode path possibly caused by needle insertion or rat movement. Bleeding was observed in tumour core at treatment location and muscle contractions were also evident and overcome with muscle relaxants.^[^ ^216^ ^]^ Drug choice for BBB disruption requires careful deliberation since highly toxic agents delivered intravenously will have wider access to penetrate and damage healthy brain tissue.

NsPEF can rapidly depolarize neurons 1 ms after application and are at risk of evoking action potentials.^[^ ^98^ ^]^ Careful consideration of protocols in particularly, pulsing durations, to allow for the response of voltage‐gated channels and reduce the likelihood of neuronal firing are encouraged.

While IRE is considered non‐thermally mediated, in vitro models have shown that for pulse durations on the order of 1µs are thermally mediated.^[^ ^112^ ^]^ Real‐time temperature monitoring during IRE applications will be a necessity to ensure that protein denaturation, oedema and seizures are avoided. Early pre‐clinical validation of IRE in tumour free canine models indicated complications of oedema, seizures and bleeding (due to needle insertion) which were overcome with corticosteroids and a hyperosmolar agent (oedema) or prophylactic anticonvulsant (seizures).^[^ ^113^ ^]^ Furthermore, in canine models with spontaneous occurring gliomas, IRE treatment was well tolerated and tumours were successfully ablated with adjunctive radiotherapy, anti‐oedema, and anti‐seizure treatment.^[^ ^116^ ^]^ In a later study involving 7 canines with glioma, one canine experienced severe cerebral oedema due to excessive field strengths.^[^ ^118^ ^]^ This outcome stresses the importance of pre‐operation planning and the demand for real‐time field monitoring in tissue to ensure safe delivery.

Treatment with H‐FIRE improved the outcomes observed in some IRE models. First, the protocol does not induce muscle contractions,^[^ ^122^, ^123^ ^]^ it has theoretically been suggested to permeabilize but not excite cells,^[^ ^97^ ^]^ and is synchronized with cardiac rhythm.^[^ ^125^ ^]^ Pre‐clinical studies of H‐FIRE indicated no adverse post‐operative effects attributed to the therapy.^[^ ^137^ ^]^

The external application of TTFs have indicated dermatologic adverse events in 16% of those enrolled in the phase III trial. This due to the contact electrodes with the skin causing irritation and rash which can be overcome with topical or oral antibiotics, topical corticosteroids.^[^ ^260^ ^]^

Finally, in pre‐clinical models of IMT, one animal died 3 days after IMT initiation, however, histological examination excluded the complication to be related to IMT treatment since there was no evidence of electrolysis or hematoma.^[^ ^172^ ^]^

It is evident that the most serious risks include oedema and seizures which have improved as techniques have been developed and can be maintained with appropriate supporting drugs. It is important that these techniques continue to minimize any associated risks as they transition from benchtop to clinic.

### 7.6 Multi‐Modality Integration—A Promising Approach

Due to the multi‐faceted nature of GBM, it seems obvious to approach treatment with a multi‐modal solution. In the presented findings, following surgical resection and radiotherapy, the addition of TTFs with maintenance TMZ resulted in greatest patient outcomes compared TMZ alone.^[^ ^152^ ^]^ ECT when used in the context of BBB disruption would allow for substantial trials of drug repurposing and targeting in GBM. Several approaches to drug repurposing for GBM have been recently reviewed,^[^ ^261^ ^]^ some of which include Chloroquine, an antimalarial agent found to increase chemosensitivity to TMZ,^[^ ^262^ ^]^ Naringenin, a bioactive flavonoid which has shown to eliminate migration and invasion in GBM,^[^ ^263^ ^]^ and Phloroglucinol a phenolic derivative and natural phlorotannin component of brown algae has suggested to inhibit proliferation.^[^ ^261^ ^]^ Additionally, Bevacizumab, Disulfiram and platinum‐based therapeutics are all considered promising for drug re‐purposing to treat gliomas and for potential administration alongside the electrotherapy techniques discussed. Combined electroporation and local drug delivery devices with adjacent or integrated ion‐pumps (i.e., convection enhanced delivery,^[^ ^264^ ^]^iontronic pumps^[^ ^265^ ^]^) to the working electrode administered under appropriate conditions, localized delivery followed by electroporation or TTFs, could also allow for delivery of chemotherapies that have limited BBB perfusion to be trialed. Integrating a small, separately controlled light source (i.e., Light emitting diode or optical fiber) to an implantable electrotherapy device could provide options for high‐resolution photodynamic therapy.^[^ ^266^ ^]^ These device modifications would provide a multifunctional treatment regime that could substantially impact the current standard of care.

## 8 Final Remarks

Glioblastoma is exceptionally complex and currently incurable. Several therapeutic options are yet to significantly advance the standard of care. Electrotherapy presents a unique therapeutic strategy but must look at addressing the complex biological challenges that currently make the disease so difficult to treat in the first place. In order to progress interdisciplinary research in this field we must first address the following: 1) The vast heterogeneity displayed by GBM is in itself a major problem when treating the disease. The presence of cells capable of repopulating the tumour heterogeneity post‐treatment and aggressively invasive cells means that current treatment cannot prevent the inevitable recurrence. Treatments cannot be expected to rise to this challenge if the challenge itself is not understood, thus further research into the mechanisms of heterogeneity are vital if novel treatments are going to be pursued. 2) While electrotherapy techniques show promise, there remains a gap of addressing the biological complexities. How do PEFs affect recurrence? Is there a neuro‐immune response? What happens to neurons in the micro‐environment and surrounding parenchyma? What is the efficacy if it is combined with TMZ, or other chemotherapies? How does it compare to the current clinical management? How does it differ between varying samples of GBM and in vivo? For TTFs, how does this technique alter the membrane potential, and thus is there a better way to apply the stimulus? Does this better explain the target mechanism, and could this reduce apprehension in the field? How does it affect the immune response? More generally, how do electromagnetic fields affect neoplastic progression and migration? We need to understand how we can use one or multiple techniques in a combined manner to both understand and treat GBM. 3) A universal approach to describe the applied electrical stimulus in electrotherapy. Currently, there is no generic ways of explaining what parameters are used in an experiment (whether it be in vitro, in vivo or in silico) which makes comparing data and reproducibility exhausting. We suggest in future a better characterized setup where all methods should include the current density of the applied stimulus (taking the integral of the active phase) such that any applied stimulus can be scaled accordingly thus compared accurately. Authors should be clear whether or not their method uses direct, capacitive, or inductive coupling (or combinations of), In addition, electrode characterization including impedance frequency response and would be advantageous. 4) Safety, TTFs excluded, all other electrotherapies reviewed here are in vitro or vivo validation in the brain. Electroporation techniques particularly need sufficient evidence that demonstrates the safety of high field strengths in the brain where current concerns raised in Section 7.5. are addressed.

It is clear that the techniques described in this review present valuable strategies for advanced treatment of glioblastoma. We encourage all the scientists working on electrotherapies to acquire an interdisciplinary approach, marrying the technological input with the biological knowledge to further advance PEFs‐ and TTFs‐based therapies to create efficient and robust methods for tackling the most aggressive type of primary brain tumours.

## Conflict of Interest

The authors declare no conflict of interest.

## Author Contributions

E.P.W.J., A.F., and M.G. contributed to the content and figures. E.P.W.J. and A.F. contributed to final editing and preparing the manuscript for submission. All authors contributed to drafting the outline and editing the manuscript.
